# FERONIA and microtubules independently contribute to mechanical integrity in the *Arabidopsis* shoot

**DOI:** 10.1371/journal.pbio.3001454

**Published:** 2021-11-12

**Authors:** Alice Malivert, Özer Erguvan, Antoine Chevallier, Antoine Dehem, Rodrigue Friaud, Mengying Liu, Marjolaine Martin, Théophile Peyraud, Olivier Hamant, Stéphane Verger

**Affiliations:** Laboratoire de Reproduction et Développement des Plantes, Université de Lyon, UCB Lyon 1, ENS de Lyon, INRAE, CNRS, Lyon, France; UCSD, UNITED STATES

## Abstract

To survive, cells must constantly resist mechanical stress. In plants, this involves the reinforcement of cell walls, notably through microtubule-dependent cellulose deposition. How wall sensing might contribute to this response is unknown. Here, we tested whether the microtubule response to stress acts downstream of known wall sensors. Using a multistep screen with 11 mutant lines, we identify FERONIA (FER) as the primary candidate for the cell’s response to stress in the shoot. However, this does not imply that FER acts upstream of the microtubule response to stress. In fact, when performing mechanical perturbations, we instead show that the expected microtubule response to stress does not require FER. We reveal that the *feronia* phenotype can be partially rescued by reducing tensile stress levels. Conversely, in the absence of both microtubules and FER, cells appear to swell and burst. Altogether, this shows that the microtubule response to stress acts as an independent pathway to resist stress, in parallel to FER. We propose that both pathways are required to maintain the mechanical integrity of plant cells.

## Abstract 2

In all living organisms, cells must resist mechanical stress to survive. This study of the model plant Arabidopsis reveals that the candidate cell wall mechanoreceptor FERONIA and microtubules independently contribute to this mechanical feedback.

## Introduction

All living organisms use mechanical forces as instructive cues during their development [1,2]. They also share a common mechanical property: Cells are pressurized by osmotic pressure and thus experience cortical tension. Osmotic pressure in plants is several orders of magnitude higher than that of animal cells, and it is counterbalanced by stiff cell walls [3]. Regulating the mechanical properties of cell walls, through the perception of wall tension and integrity, is thus crucial for plant growth and development [4,5].

Plant cell walls are composed of load-bearing cellulose microfibrils, tethered by a matrix made of polysaccharides and structural proteins [6,7]. The deposition of cellulose microfibrils is generally guided by cortical microtubules [8]. Beyond the average stiffness, the orientation of cellulose microfibrils controls the mechanical anisotropy of the wall. There is now ample evidence showing that cortical microtubules align with maximal tensile stress directions in the wall [9,10–13]. This provides a feedback loop in which shape and growth, whether at the individual cell or whole organ scale, prescribes a pattern of stress, to which cells resist by reinforcing their walls along maximal tensile stress directions [12,14].

Although they are significantly less stiff, other wall components contribute to wall properties. In particular, pectins can partially rescue defects in cellulose synthesis in young cell walls. For instance, isoxaben treatment, which inhibits cellulose deposition through the internalization of CESA complexes, leads to thicker walls that are enriched in pectin [15]. Similarly, in young hypocotyls, pectin polarities precede the formation of mechanically anisotropic walls [16]. In contrast to cellulose deposition, pectin, as well as all other matrix components, are secreted to the cell wall [6,7]. Therefore, in principle, this provides an alternative mechanism for the cell to resist wall tension or damage. As for microtubules, the related mechanotransduction pathway is largely unknown. Yet, over the past decade, *Catharanthus roseus* Receptor-Like Kinases (CrRLKs) have emerged as key players. Although the link with mechanical stress remains to be formally established, THESEUS1 (THE1) has been implicated in the wall integrity pathway [17–19]. Based on defective root growth behavior on stiff interface, calcium signaling, pH response, and *TOUCH* gene expression, FERONIA (FER) has emerged as a candidate mechanosensor [20]. FER can sense the status of the cell wall, notably when salinity rises, through pectin binding [21]. It was recently proposed that FER also monitors microtubule behavior through a cascade involving Rho GTPases (ROP6) and the microtubule severing protein katanin [22]. Here, through a reverse genetic screen on wall sensors and using a suite of mechanical tests, we show that our best wall sensor candidate FER is not required for the microtubule response to stress, further suggesting that the microtubule response to stress can be more autonomous than anticipated. We also reveal that FER-dependent wall integrity pathway depends on wall tension and that both FER and the microtubule response to stress contribute to wall integrity.

## Results

### Altered pavement cell shape as a proxy for defective response to mechanical stress

**Fig 1:**
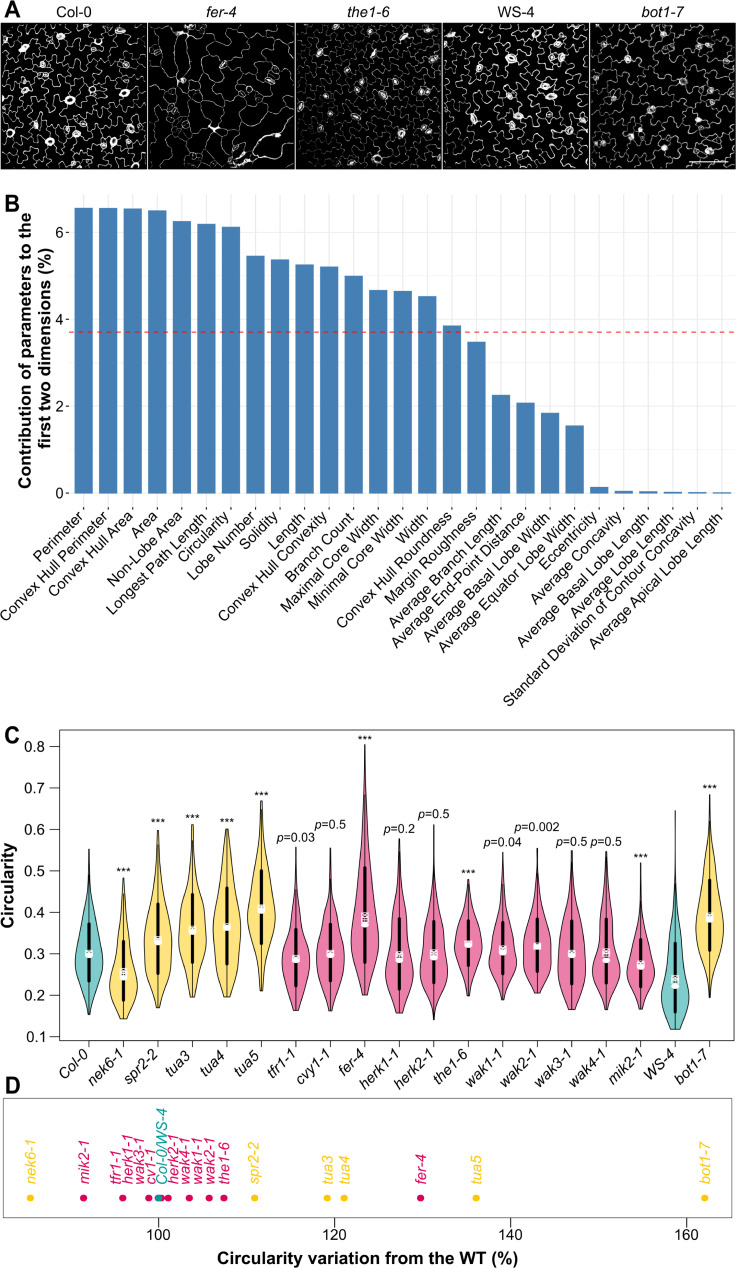
Pavement cell shape in receptor-like kinase mutants. **(A)** Representative images of *Col-0*, *WS-4*, *fer-4*, *the1-6*, and *bot1-7* pavement cells. Samples were PI stained, and cell contours were extracted with MorphoGraphX and projected in 2D. Scale = 100 μm. **(B)** Relative contribution of 27 shape descriptors to pavement cell shape, as assessed by principal component analysis. **(C)** Circularity (violin plots) of pavement cells and *p*-values of Dunn tests for the WT (in blue, *Col-0* and *WS-4*), for the microtubule regulator mutants (in orange, *nek6-1*, *spr2-2*, *tua3*, *tua4*, *tua5*, and *bot1-7*) and the receptor-like kinase mutants (in pink, *tfr1-1*, *cvy1-1*, *fer-4*, *herk1-1*, *herk2-1*, *the1-4*, *the1-6*, *wak1-1*, *wak2-1*, *wak3-1*, *wak4-1*, and *mik2-1*). **(D)** Percentage of increase or decrease in pavement cell circularity from the WT (in blue, *Col-0* and *WS-4*), for the microtubule regulator mutants (in orange, *nek6-1*, *spr2-2*, *tua3*, *tua4*, *tua5*, and *bot1-7*) and the receptor-like kinase mutants (in pink, *tfr1-1*, *cvy1-1*, *fer-4*, *herk1-1*, *herk2-1*, *the1-4*, *the1-6*, *wak1-1*, *wak2-1*, *wak3-1*, *wak4-1*, and *mik2-1*). All underlying data can be found in S1 Data. PI, propidium iodide; WT, wild type.

We first used a morphometric proxy to test the involvement of wall sensors in the microtubule response to stress. The jigsaw puzzle shape of *Arabidopsis* pavement cells has been proposed to be actively maintained and amplified by the microtubule response to mechanical stress. Indeed, necks in such cells prescribe highly anisotropic tensile stresses locally, to which microtubule arrays and thus cellulose deposition align [14,23,24]. We reasoned that the shape of pavement cells could be used in a mutant screen as a proxy for defects in that mechanical feedback loop. In past research, such screens have targeted the intracellular biochemical cues behind cell–cell coordination [25] and the contribution of cell wall properties in cell shape [26]. Whether wall sensors are involved in pavement cell shape remains ill described. Here, we focused on mutants impaired in receptor-like kinases that are highly expressed in the epidermis and aerial parts of the plant during early development and that exhibit an established link with the cell wall (and their closest homologs), namely *feronia (fer)*, *theseus1 (the1)*, *theseus1/feronia-related1* (*tfr1*; *at5g24010)*, *curvy1* (*cvy1)*, *hercules receptor kinase 1* (*herk1)*, *herk2*, *mdis1-interacting receptor-like kinase2 (mik2-1)*, *wall-associated kinase 1 (wak1)*, *wak2*, *wak3*, and *wak4* (see S1 Table). We imaged and quantified the pavement cell shapes in receptor-like kinase candidate mutants, with the aim to select the ones with the strongest cell shape defects (Fig 1A).

To do so, we extracted the epidermal signal and used PaCeQuant to segment pavement cells and measure 27 shape descriptors (see Materials and methods [27] (S1A Fig)). To select the most discriminating PaCeQuant shape descriptor, we first performed a principal component analysis on our data. We compared the contribution of each parameter to the 2 axes with the most variation (Fig 1B). The cell perimeter was first, followed by the convex hull perimeter, the convex hull area, the cell area, the nonlobe area, and the longest path length, then by circularity (for relevant definition, see S1B Fig).

To confirm that such shape descriptors are pertinent, and knowing that cortical microtubules are well-known pavement cell shape regulators, we used mutants with microtubule defects as positive controls. We performed the same analysis on 2 loss-of-function mutants: *nek6* is impaired in a tubulin kinase which depolymerizes microtubules, and the mutant exhibits an enhanced microtubule response to stress [28]; *bot1* is impaired in the microtubule severing protein katanin and exhibits a reduced response to stress [29]. We also included lines with tubulin mutations affecting microtubule dynamics (*tua3*^*D205N*^, *tua4*^*S178Δ*^, and *tua6*^*A281T*^ referred as *tua3*, *tua4*, and *tua5* in the following [30,31]) and *spr2* with a reported enhanced cortical microtubule response to stress [32] but also ambivalent regulatory role in microtubule severing depending on tissue [33–35]. We found that cell perimeter, convex hull perimeter, convex hull area, cell area, and the longest path length descriptors were not sufficient to discriminate the pavement cell shape phenotype of those microtubules regulators (cell perimeter: *p*_*spr2-2*_ = 0.07; *p*_*tua3*_ = 0.018; *p*_*tua4*_ = 0.4; convex hull perimeter: *p*_*tua3*_ = 0.39; *p*_*tua5*_ = 0.011; convex hull area: *p*_*tua3*_ = 0.5; *p*_*tua5*_ = 0.03; cell area: *p*_*tua3*_ = 0.06; *p*_*tua5*_ = 0.07; longest path length: *p*_*tua3*_ = 0.28; *p*_*tua4*_ = 0.1; n_*spr2-2*_ = 262; n_*tua3*_ = 204; n_*tua4*_ = 230; n_*tua5*_ = 297; n_*Col-0*_ = 428). By contrast, all microtubule regulator mutant lines tested exhibited a defect in nonlobe area and circularity (nonlobe area: *p*_*nek6-1*_ = 0.009; *p*_*spr2-2*_ < 10^−3^; *p*_*tua3*_ = 0.003; *p*_*tua4*_ < 10^−3^; *p*_*tua5*_ < 10^−3^; *p*_*bot1-7*_ < 10^−3^; circularity: *p*_*nek6-1*_ < 10^−3^; *p*_*spr2-2*_ < 10^−3^; *p*_*tua3*_ < 10^−3^; *p*_*tua4*_ < 10^−3^; *p*_*tua5*_ < 10^−3^; *p*_*bot1-7*_ < 10^−3^, n_*nek6-1*_ = 183; n_*spr2-2*_ = 262; n_*tua3*_ = 204; n_*tua4*_ = 230; n_*tua5*_ = 297; n_*bot1-7*_ = 252; n_*Col-0*_ = 428; n_WS-4_ = 294, Fig 1C, S1C Fig).

A total of 9 of the 11 receptor-like kinase mutants exhibited a nonlobe area significantly different from that of the wild type (WT). *tfr1-1*, *fer-4*, *herk1-1*, *herk2-1*, and *wak4-1* displayed a nonlobe area significantly higher than that of the WT (*p*_*tfr1-1*_ < 10^−3^; *p*_*fer-4*_ < 10^−3^; *p*_*herk1-1*_ < 10^−3^; *p*_*herk2-1*_ < 10^−3^; *p*_*wak4-1*_ = 0.007; n_*tfr1-1*_ = 244; n_*fer-4*_ = 321; n_*herk1-1*_ = 225; n_*herk2-1*_ = 238; n_*wak4-1*_ = 204; n_*Col-0*_ = 428, S1C Fig), while the nonlobe area of *cvy1-1*, *the1-6*, *wak1-*,*1* and *mik2-1* was significantly lower than that of the WT (*p*_*cvy1-1*_ < 10^−3^; *p*_*the1-6*_ < 10^−3^; *p*_*wak1-1*_ < 10^−3^; *p*_*mik2-1*_ < 10^−3^; n_*cvy1-1*_ = 277; n_*the1-6*_ = 174; n_*wak1-1*_ = 295; n_*mik2-1*_ = 177; n_*Col-0*_ = 428). Only *wak2-1* and *wak3-1* displayed nonlobe area values that were nonsignificantly different from that of the WT (*p*_*wak2-1*_ = 0.1; *p*_*wak3-1*_ = 0.03; n_*wak2-1*_ = 271; n_*wak3-1*_ = 202; n_*Col-0*_ = 428). Thus, nonlobe area is not a discriminant parameter in our screen. We decided to study the next most variable parameter with defects in known microtubule regulator lines—circularity—for the rest of the analysis, justifying a posteriori a common choice in the literature on pavement cell shape [36].

Among the receptor-like kinase mutants tested, the pavement cells in *fer-4*, *the1-6*, and *wak2-1* were significantly more circular than the WT supporting the hypothesis that the corresponding proteins could contribute to the microtubule response to stress in pavement cells (*p*_*fer-4*_ < 10^−3^; *p*_*the1-6*_ < 10^−3^; *p*_*wak2-1*_ = 0.002; n_*fer-4*_ = 321; n_*the1-6*_ = 174; n_*wak2-1*_ = 271; n_*Col-0*_ = 428; Fig 1C). *wak1-1* also exhibited increased pavement cell circularity, albeit much less significantly (*p*_*wak1-1*_ = 0.04; n_*wak1-1*_ = 295; n_*Col-0*_ = 428). Only *mik2-*1 exhibited a significantly decreased pavement cell circularity (*p*_*mik2-1*_ < 10^−3^; n_*mik2-1*_ = 177; n_*Col-0*_ = 428), while *tfr1-1* exhibited a tendency toward a decreased pavement cell circularity (*p*_*tfr1-1*_ = 0.03; n_*tfr1-1*_ = 244; n_*Col-0*_ = 428). Pavement cells in all the other receptor-like kinase mutants (*cvy1-1*, *herk1-1*; *herk2-1*; *wak3-1*; *wak4-1*) displayed a circularity comparable to that of the WT (p_*cvy1-1*_ = 0.5; *p*_*herk1-1*_ = 0.2; *p*_*herk2-1*_ = 0.47; *p*_*wak3-1*_ = 0.47; *p*_*wak4-1*_ = 0.5; n_*cvy1-1*_ = 277; n_*herk1-1*_ = 225; n_*herk2-1*_ = 238; n_*wak3-1*_ = 202; n_*wak4-1*_ = 204; n_*Col-0*_ = 428). To distinguish the relative contributions of the most affected mutants, we quantified the deviation of circularity from the WT. Among all the receptor-like kinases tested, the candidate mutants with the largest defect in circularity when compared to the WT were *fer-4* (consistent with previously published results, [37]) and, to a lesser extent, *the1-6* (Fig 1D). *fer-4* cells were 30% more circular and *the1-6* cells were 7% more circular than the WT, values that were comparable to that of microtubule regulator mutants, such as *spr2-2* or *bot1-7* (Fig 1D).

Note that the same trend was obtained when considering solidity, another parameter often used to characterize lobe formation [38] (S1D Fig). Note also that while circularity and solidity can be impacted when stress response levels change, they do not necessarily directly scale with stress response level. Different shapes can lead to similar circularity and solidity values (e.g., see last figure); nevertheless, this cell shape-based screening allowed us to identify the RLK mutants with the most affected pavement cell shape, possibly through a defective microtubule response to stress. Because *fer-4* stands out, this first screening suggests that FER could play a role in the microtubule response to mechanical stress.

### Differential response of receptor-like kinase mutants to isoxaben

To challenge the results from this initial screen, we next used a well-established protocol to mechanically perturb cell walls. Isoxaben inhibits the biosynthesis of cellulose [39], and thus weakens the wall. In past work, such treatment were shown to induce a hyperalignment of cortical microtubules at the shoot apical meristem and in cotyledon pavement cells [14,40], consistent with a response to increased tensile stress levels in the cell wall. Note that isoxaben can also trigger other responses, including reactive oxygen species (ROS) production, lignification, and changes in gene expression [17]. Thus, depending on time and dose, isoxaben may also ultimately reduce stress level [41]. Here, we use this drug as a screening tool, complementary to the pavement cell shape screen, to identify mutants insensitive or hypersensitive to mechanical perturbation and which are thus likely to be defective in mechanosensing.

**Fig 2:**
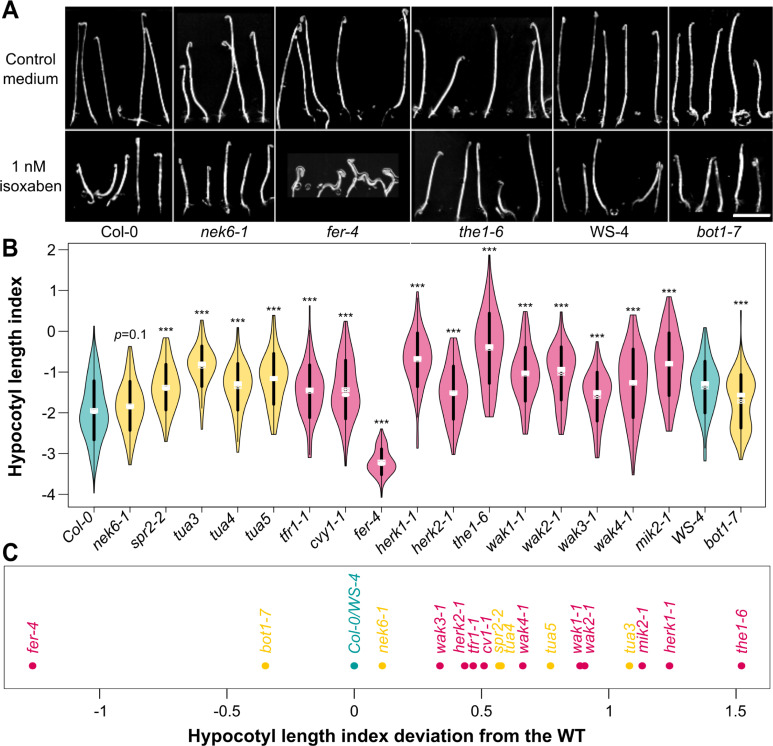
Impact of isoxaben on hypocotyl length in receptor-like kinase mutants. **(A)** Representative images of *Col-0*, *WS-4*, *fer-4*, *the1-*,*6* and *bot1-7* etiolated seedlings grown with or without 1 nM isoxaben. Scale = 1 cm. **(B)** Hypocotyl length index (violin plot): distribution of isoxaben-grown hypocotyl length, normalized relative to the DMSO-grown ones. *p*-values of Wilcoxon–Mann–Whitney test for the WT (in blue, *Col-0* and *WS-4*), the microtubule regulator (in orange, *nek6-1*, *spr2-2*, *tua3*, *tua4*, *tua5*, and *bot1-7*), and the receptor-like kinase mutants (in pink, *tfr1-1*, *cvy1-1*, *fer-4*, *herk1-1*, *herk2-1*, *the1-4*, *the1-6*, *wak1-1*, *wak2-1*, *wak3-1*, *wak4-1*, and *mik2-1*). **(C)** Deviation of hypocotyl length index. The WT accessions (*Col-0* and *WS-4*) are labeled in blue. The microtubule regulator mutants (*nek6-1*, *spr2-2*, *tua3*, *tua4*, *tua5*, and *bot1-7*) are labeled in orange. The receptor-like kinase mutants (*tfr1-1*, *cvy1-1*, *fer-4*, *herk1-1*, *herk2-1*, *the1-4*, *the1-6*, *wak1-1*, *wak2-1*, *wak3-1*, *wak4-1*, and *mik2-1*) are labeled in pink. All underlying data can be found in S2 Data. WT, wild type.

We grew the seedlings in a medium containing 1 nM isoxaben or the same volume of DMSO, in the dark (Fig 2A). We then measured the length of the etiolated hypocotyls 4 days after germination. After isoxaben treatment, 4-day-old WT seedlings exhibited a shorter hypocotyl (by 41% for *Col-0*, 34% for *WS-4*, n_*Col-0* DMSO_ = 281, n_*Col-0* iso_ = 271, n_*WS-4* DMSO_ = 127, n_*WS-4* iso_ = 129). To compare WT and mutants, we normalized the obtained distribution of lengths to the same mean and standard deviation as the control, thus providing a hypocotyl length index (Fig 2B, S2 Fig). Treated mutants with a relatively shorter hypocotyl than the treated WT were labeled more sensitive to isoxaben, whereas treated mutants with a relatively longer hypocotyl than the treated WT were labeled less sensitive than the WT.

A total of 10 out of the 11 receptor-like kinase mutants studied were less sensitive than the WT (*tfr1-1*, *cvy1-1*, *herk1-1*, *herk2-1*, *the1-6*, *wak1-1*, *wak2-1*, *wak3-1*, *wak4-1*, *mik2-1*; *p*_*tfr1-1*_ < 10^−3^; *p*_*cvy1-1*_ < 10^−3^; *p*_*herk1-1*_ < 10^−3^; *p*_*herk2-1*_ < 10^−3^; *p*_*the1-6*_ < 10^−3^; *p*_*wak1-1*_ < 10^−3^; *p*_*wak2-1*_ < 10^−3^; *p*_*wak3-1*_ < 10^−3^; *p*_*wak3-1*_ < 10^−3^; *p*_*mik2-1*_ < 10^−3^; n_*tfr1-1* DMSO_ = 80; n_*tfr1-1* iso_ = 83; n_*cvy1-1* DMSO_ = 109; n_*cvy1-1* iso_ = 115; n_*herk1-1* DMSO_ = 82; n_*herk1-1* iso_ = 70; n_*herk2-1* DMSO_ = 88; n_*herk2-1* iso_ = 106; n_*the1-6* DMSO_ = 57; n_*the1-6* iso_ = 91; n_*wak1-1* DMSO_ = 116; n_*wak1-1* iso_ = 89; n_*wak2-1* DMSO_ = 63; n_*wak2-1* iso_ = 78; n_*wak3-1* DMSO_ = 99; n_*wak3-1* iso_ = 89; n_*wak4-1* DMSO_ = 103; n_*wak4-1* iso_ = 86; n_*mik2-1* DMSO_ = 84; n_*mik2-1* iso_ = 96; n_*Col-0* DMSO_ = 281; n_*Col-0* iso_ = 271), whereas *fer-4* was significantly more sensitive (*p*_fer-4_ < 10^−3^; n_*fer-4* DMSO_ = 104; n_*fer-4* iso_ = 90) than the WT (Fig 2B). When plotting the deviation of each mutant from the WT phenotype, it appeared that among all the receptor-like kinase tested, *fer-4* and *the1-6* were the most affected mutants in their response to isoxaben, albeit in opposite trend: *fer-4* hypocotyl were more sensitive to isoxaben, whereas *the1-*6 were less sensitive to isoxaben (Fig 2C). These results indicate that all the receptor-like kinases tested might be involved in the cellular response to a mechanical stress, with *FER* and *THE1* having the most clear-cut, and opposing, response.

To check whether these defects could be related to the microtubule response to stress, we performed the same analysis on microtubule regulator mutants. *bot1-7* (in *WS-4* ecotype) was more sensitive to the isoxaben treatment than the WT (*p*_*bot1-7*_ < 10^−3^; n_*bot1-7* DMSO_ = 99; n_*bot1-7* iso_ = 96; n_WS-4 DMSO_ = 127; n_WS-4 iso_ = 129) and thus fell in the same cluster as *fer-4*. The *nek6-1* mutant exhibited the same isoxaben sensitivity as the WT (*p*_*nek6-1*_ = 0.1; n_*nek6-1* DMSO_ = 91; n_*nek6-1* iso_ = 104; n_*Col-0* DMSO_ = 281; n_*Col-0* iso_ = 271) (Fig 2B). The *spr2-2* mutant was significantly less sensitive than the WT (p_*spr2-2*_ < 10^−3^; n_*spr2-2* DMSO_ = 99; n_*spr2-2* iso_ = 85; n_*Col-0* DMSO_ = 281; n_*Col-0* iso_ = 271) and thus fell in the same cluster as *the1-6*. Last, *tua3*, *tua4*, and *tua5* were significantly less sensitive than the WT (*p*_*tua3*_ < 10^−3^; *p*_*tua4*_ < 10^−3^; *p*_*tua5*_ < 10^−3^; n_*tua3* DMSO_ = 93; n_*tua3* iso_ = 90; n_*tua4* DMSO_ = 83; n_*tua4* iso_ = 108; n_*tua5* DMSO_ = 72; n_*tua5* iso_ = 66; n_*Col-0* DMSO_ = 281; n_*Col-0* iso_ = 271).

Altogether, our primary screening approach using both pavement cell shape analysis and isoxaben sensitivity test as a combined proxy for the microtubule response to mechanical stress highlights FER as a primary candidate. So far, our data are also consistent with the proposed scenario in which FER and katanin belong to the same pathway [22], but these remain primary screening approaches that do not directly test the involvement of our main candidate in the microtubule response to mechanical stress. Based on this screening, in the following we decided to focus on FER to investigate its involvement in the mechanical integrity of the shoot and to directly test its contribution to the microtubule response to mechanical stress.

### Rescue test: Decreasing the matrix potential of the growth medium rescues the burst cell phenotype in *feronia*

We first decided to further investigate the isoxaben hypersensitivity phenotype of *fer-4*. It is commonly believed that WT seedlings display a shorter hypocotyl on isoxaben because cell wall defects are perceived and compensated through wall reinforcement, ultimately leading to reduced growth. Both wall reinforcement and reduced growth would prevent the cells from bursting. This notably builds on the analysis of *the1* mutant, which exhibits a longer hypocotyl than the WT on isoxaben because of the lack of wall sensing [17]. In *fer*, the hypocotyl is in contrast even shorter than the WT. Thus, either wall sensing is enhanced in *fer* thus strongly repressing growth, or, by contrast, wall sensing is impaired in *fer*, even more than in *the1*, and cells burst before walls can even be reinforced. In the rest of the article, we focus the analysis of the *fer-4* allele, but similar results could be obtained in *fer-2* (S3 Fig).

**Fig 3:**
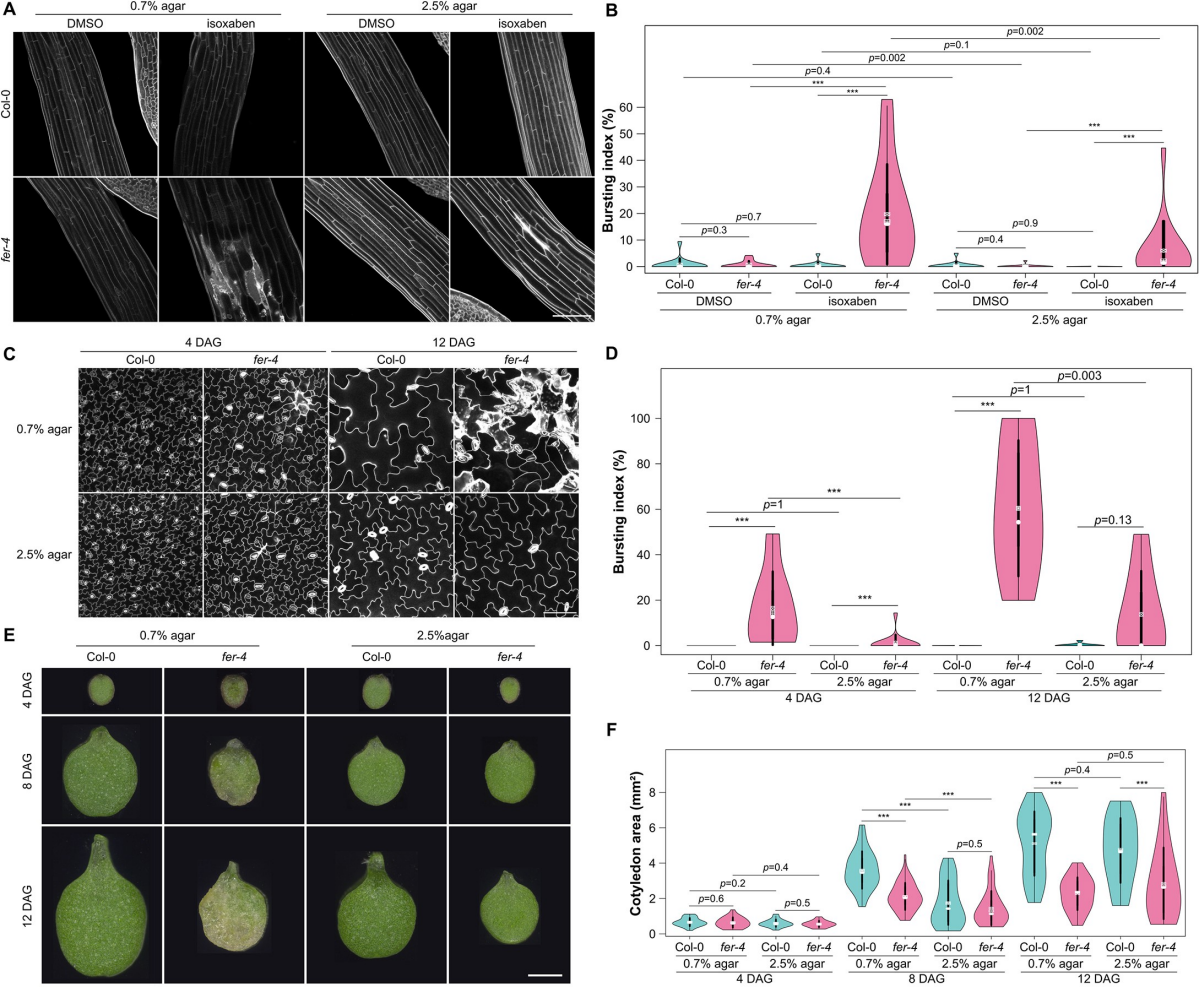
The *fer* phenotype can be partially rescued on 2.5% agar. **(A)** Representative confocal images of Col-0 and *fer-4* etiolated hypocotyls, from seedlings grown on a medium containing 1 nM isoxaben with 0.7% or 2.5% agar (propidium iodide staining). Scale = 100 μm. **(B)** Bursting index (violin plot) and *p*-values of Wilcoxon–Mann–Whitney tests in *Col-0* and *fer-4* etiolated hypocotyls grown on a medium containing 1nM isoxaben with 0.7% or 2.5% agar. **(C)** Representative confocal images of *Col-0* and *fer-4* pavement cells, from seedlings grown on a medium containing 0.7% or 2.5% agar at t = 4 DAG and t = 12 DAG (propidium iodide staining). Scale = 100 μm. **(D)** Bursting index (violin plot) and *p*-values of Wilcoxon–Mann–Whitney tests in *Col-0* and *fer-4* pavement cells, from seedlings grown on a medium containing 0.7% or 2.5% agar at t = 4 DAG and t = 12 DAG. **(E)** Representative images of *Col-0* and *fer-4* cotyledons, grown on a medium containing 0.7% or 2.5% agar at t = 4 DAG, t = 8 DAG, and t = 12 DAG. Scale = 1 mm. **(F)** Cotyledon area (violin plot) and *p*-values of Wilcoxon–Mann–Whitney tests in *Col-0* and *fer-4* seedlings, grown on a medium containing 0.35%, 0.7%, 1.25%, or 2.5% agar at t = 4 DAG, t = 8 DAG, and t = 12 DAG. All underlying data can be found in S3 Data.

When looking closely at *fer-4* hypocotyls grown on isoxaben and stained with propidium iodide (PI), we observed many dead cells, as revealed by PI staining (Fig 3A). We calculated a bursting index, i.e., the percentage of burst cell area over total area of a field of epidermal cells in a given image. We found the bursting index to be more than one order of magnitude higher in *fer-4* isoxaben-grown hypocotyls (20 ± 18%) than in the WT (0 ± 1%, *p* < 10^−3^; n_*Col-0*,iso,0.7%agar_ = 16; n_*fer-4*,iso,0.7%agar_ = 16, Fig 3B). This observation thus seems consistent with the latter scenario.

To test this hypothesis further, we reasoned that lowering the water potential in the medium should reduce water intake for seedlings, reduce tensile stress level [13], and in the end, reduce the number of burst cells. We thus tested different agar concentration in the medium and analyzed the impact on *fer-4* phenotype. When increasing the agar concentration to 2.5%, the bursting index in hypocotyls was reduced by 70% in isoxaben-grown seedlings, supporting our hypothesis (p = 0.002; n_fer-4,isoxaben,0.7%agar_ = 16; n_fer-4,isoxaben,2.5%agar_ = 16, Fig 3A and 3B).

We then took a closer look at the pavement cells of *fer-4* cotyledons grown in a medium containing a standard concentration of agar (0.7%) and over time. When looking at orthogonal sections, the outer cell wall appeared very deformed, consistent with cell swelling in *fer-4*, when compared to WT (S4 Fig). This geometry echoes previously observed phenotypes and is consistent with increased wall stretch in *fer* [20,21,42].

In these growth conditions, the mechanical integrity of the cell was not maintained in *fer*: Many burst cells were present, as marked by a strong PI coloration (Fig 3C, S4 Fig). The bursting index increased over time, from 16 ± 16% at 4 DAG (days after germination) to 60 ± 30% at 12 DAG. At both time points, it was significantly higher to that of the WT (*p*_4DAG,0.7%agar_ < 10^−3^; *p*_12DAG,0.7%agar_ < 10^−3^; n_*Col-0*,4DAG,0.7%agar_ = 14, n_*Col-0*,12DAG,0.7%agar_ = 9, n_*fer-4*,4DAG,0.7%agar_ = 14, n_*fer-4*,12DAG,0.7%agar_ 8; Fig 3D). To further confirm that burst cells appear in response to mechanical stress, we exposed cotyledon to an osmotic shock and monitored cell behavior. We transferred seedlings grown on 2.5% agar for 4 days to 0.35% agar, reasoning that this sudden change should challenge the *fer-4* cell walls. As predicted, many *fer-4* cells swelled and burst in the hours following the transfer (S5 Fig, S1 Movie).

Next, we reasoned that maintaining seedling on 2.5% agar might rescue the *fer-4* phenotype in cotyledons too. In these conditions, the bursting index in *fer-4* was still higher to that of the WT at 4 DAG (*p*_4DAG,2.5%agar_ < 10^−3^; n_*Col-0*,4DAG,2.5%agar_ = 15; n_*fer-4*,4DAG,2.5%agar_ = 15, Fig 3D). However, the bursting index was reduced to 1 ± 4% at 4 DAG (*p*_*fer-4*,4DA*G*_ < 10^−3^) and to 14 ± 19% at 12 DAG (*p*_*fer-4*,12DAG_ = 0.003, Fig 3D), which made it not significantly different from the WT (*p*_12DAG,2.5%agar_ = 0.13; n_*Col-0*,12DAG,2.5%agar_ = 9, n_*fer-4*,12DAG,2.5%agar_ = 7; Fig 3D). Once again, lowering the water availability for cells partially rescued the bursting cell phenotype in *fer-4*.

To check whether the apparent rescue could have a more global effect on organ shape, we measured *fer-4* cotyledon area over time on seedlings grown on media containing different agar concentrations (0.7%, 2.5%, Fig 3E and 3F). At a young stage (4 days of light), *fer-4* cotyledons had comparable area as WT ones for every agar concentration (*p*_4DAG,0.7%agar_ = 0.63; *p*_4DAG,2.5%agar_ = 0.54; n_*Col-0*,4DAG,0.7%agar_ = 38; n_*fer-4*,4DAG,0.7%agar_ = 23; n_*Col-0*,4DAG,2.5%agar_ = 35; n_*fer-4*,4DAG,2.5%agar_ = 27, Fig 3E and 3F). After 8 days of light, *fer-4* cotyledons were 41% smaller than the WT for seedling grown on a medium containing 0.7% agar (*p*_8DAG,0.7%agar_ < 10^−3^; n_*Col-0*,8DAG,0.7%agar_ = 45; n_*fer-4*,8DAG,0.7%agar_ = 56). Strikingly, *fer-4* cotyledons reached a size comparable to that of the WT for seedlings grown on 2.5% agar (*p*_8DAG,2.5%agar_ = 0.51; n_*Col-0*,8DAG,2.5%agar_ = 55; n_*fer-4*,8DAG,2.5%agar_ = 40). However, this trend was not maintained after 12 days of light (Fig 3E and 3F).

Note that in all these experiments, the aerial phenotype is rescued, even though only the roots are in contact with a medium with different agar concentrations. This is consistent with the idea that the differences in matrix potential is the cause of the rescue (and not the contact with soft or stiff agar). This is also consistent with previous results obtained in other mutants with cell wall defects, such as the *quasimodo* mutants, where it was also confirmed, using atomic force microscopy, that growth on 2.5% agar medium significantly decreased the apparent tensions in the cotyledon’s outer epidermal cell walls [13].

Thus, we find a correlation between the mechanical status of the *fer-4* cotyledons (as monitored by the bursting index) and growth. Taken together, these results suggest that the *fer-4* mutant is unable to sense the mechanical status of the tissue and that the *fer-4* phenotype reflects a passive turgor-dependent wall defect.

### Cortical microtubules orient with predicted maximal tensile stress after ablation in *fer-4*

**Fig 4:**
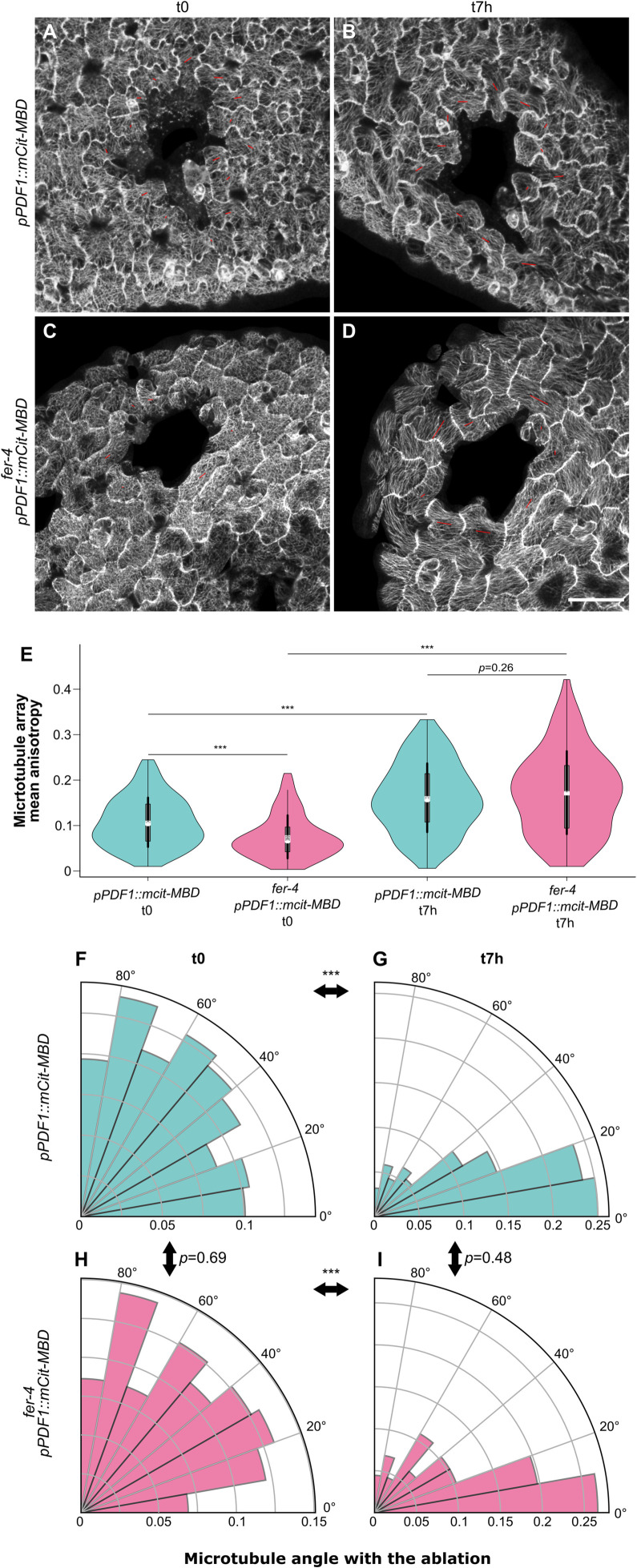
Cortical microtubule alignment in *fer* after ablation. All seedlings were grown on 2.5% agar. **(A–D)** Representative confocal images of *pPDF1*::*mCit-MBD* (A, B) and *fer-4 pPDF1*::*mCit-MBD* (C, D) pavement cells, immediately after an ablation (t0, A, C) and 7 hours later (t7h, B, D). The red bars indicate the average orientations of cortical microtubule arrays, and the length of the red bars is proportional to the anisotropy of the cortical microtubule arrays (using ImageJ FibrilTool). Scale = 50 μm. **(E)** Anisotropy (violin plot) of cortical microtubule arrays and *p*-values of Wilcoxon–Mann–Whitney tests in cells surrounding the ablation site in *pPDF1*::*mCit-MBD* and *fer-4 pPDF1*::*mCit-MBD* pavement cells, immediately after an ablation (t0) and 7 hours later (t7h). **(F–I)** Cortical microtubule orientations (polar plots) and *p*-values of Wilcoxon–Mann–Whitney tests in cells surrounding the ablation site in *pPDF1*::*mCit*-*MBD* (F, G) and *fer-4 pPDF1*::*mCit-MBD* (H, I) pavement cells, immediately after an ablation (t0, F, H)) and 7 hours later (t7h, G, I). All underlying data can be found in S4 Data.

So far, all the tests suggest that FER is a major player in the mechanical integrity the shoot, consistent with signaling and growth data obtained in the root [20] and with growth data in the hypocotyl [43]. However, it is not clear whether this involves the microtubule response to mechanical stress. To formally check this, we analyzed the behavior of cortical microtubules in response to local ablation in *fer-4*. From a mechanical standpoint, the sudden drop of turgor pressure in the ablated cells, together with epidermal tension [44], generates a circumferential tensile stress around the dead cells [12]. Such a perturbation causes the cortical microtubules to reorient in the new maximal tensile stress direction (circumferential) around the ablation [12,14]. We used the microtubule reporter *pPDF1*::*mCit-MBD* to monitor the microtubule response in *Col-0* and *fer-4* cotyledons. As a 0.7% agar medium triggers widespread cell death in *fer-4* (Fig 3C, S6A–S6D Fig), all tests were performed on 2.5% agar. We measured both the anisotropy and the average orientation of cortical microtubule arrays (relative to the ablation site) in cells surrounding the ablation (Fig 4, S7 and S8 Figs).

The anisotropy (*a*) of cortical microtubule arrays was low in the WT immediately after ablation (*a*_*Col-0*,t0_ = 0.11; n_*Col-0*,t0_ = 248). It increased by 50% 7 hours later, consistent with the co-alignment of cortical microtubules with tensile stress (*a*_*Col-0*,t7h_ = 0.16; *p* < 10^−3^; n_*Col-0*,t7h_ = 220; Fig 4E). In *fer-4*, cortical microtubule arrays were slightly less anisotropic (by 30%) than the WT at t = 0 hour (*a*_*fer-4*,t0_ = 0.075; *p* < 10^−3^; n_*fer-4*,t0_ = 174). After 7 hours, the anisotropy of cortical microtubule arrays increased by 129% and became comparable to that of the WT (*a*_*fer-4*,t7h_ = 0.17; *p* = 0.26; *n*_*fer-4*,t7h_ = 203, Fig 4E).

Immediately after the ablation, cortical microtubule arrays exhibited no preferred orientation (*o*) around the ablation, with an average angle of 47 ± 25° for the WT, which was not significantly different from that of *fer-4* (*o*_*fer-4*,t0_ = 46±25°; *p* = 0.69; Fig 4F and 4H, S7B Fig). At t0, both distributions followed a uniform law on 0 to 90° (*p*_*Col-0*,t0_ = 0.5; *p*_*fer-4*,t0_ = 0.8; Kolmogorov–Smirnov test for uniformity). After 7 hours, cortical microtubules did not follow a uniform distribution anymore (*p*_*Col-0*,t7h_ < 10^−3^; *p*_*fer-4*,t7h_ < 10^−3^; Kolmogorov–Smirnov test for uniformity) and became more circumferential around the ablation in both *Col-0* and *fer-4*, with no significant difference between the genotypes (*o*_*Col-0*,t7h_ = 28 ± 23°, *o*_*fer-4*,t7h_ = 31 ± 25°; *p* = 0.48, Fig 4G and 4I, S7 and S8 Fig).

Similar trends could be observed when using the *p35S*::*GFP-TUB* microtubule marker line: cortical microtubule orientation appeared circumferential around ablations in both WT and *fer-4* (S9 Fig). However, the diffuse fluorescent signal hindered quantitative analysis with FibrilTool. Because the microtubule response to ablation is comparable in *fer-4* and in the WT, this formally shows that the microtubule response to stress can be independent from FER.

### FER and microtubules independently contribute to the mechanical integrity of the shoot in response to mechanical stress

**Fig 5:**
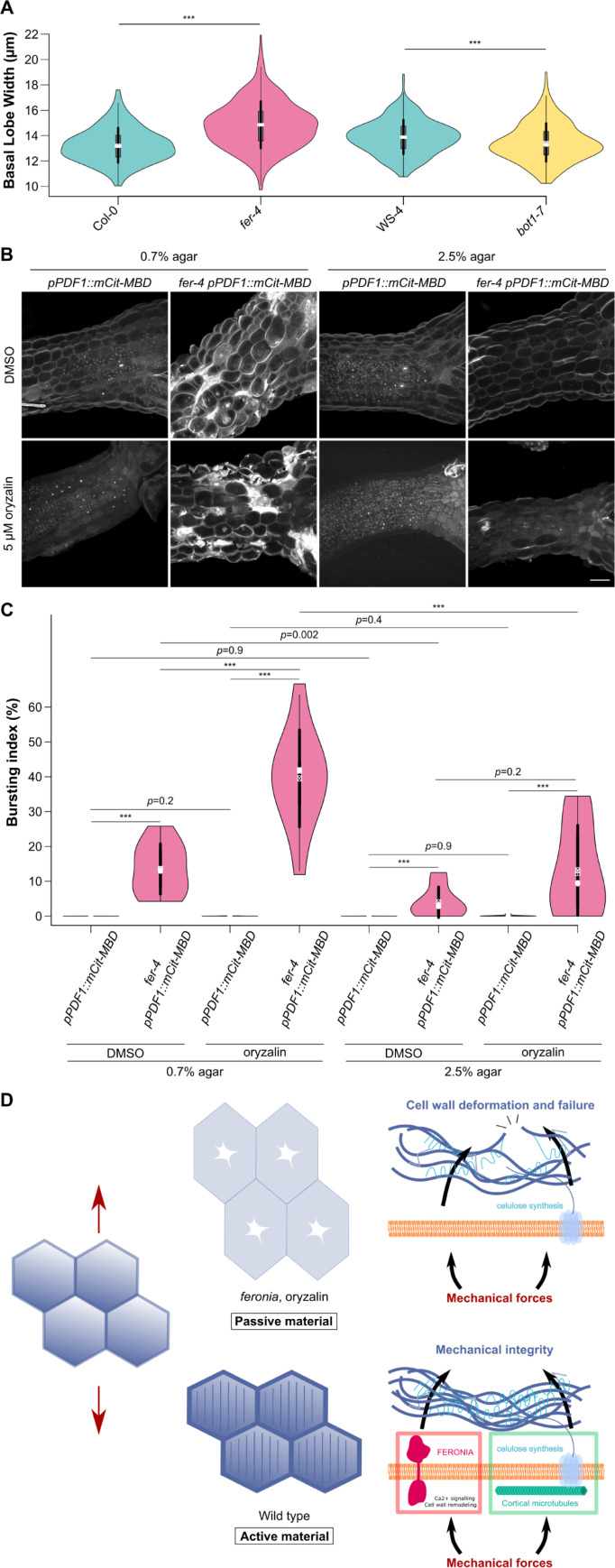
FER and microtubules independently contribute to the response to mechanical stress. **(A)** Basal lobe width (violin plot) of pavement cells and *p*-values of Dunn tests for the WT (*Col-0*, *WS-4*), katanin mutant (*bot1-7*), and *fer-4*. Seedlings were grown on 0.8% agar. **(B)** Representative confocal images of *pPDF1*::*mCit-MBD* and *fer-4 pPDF1*::*mCit-MBD* hypocotyls grown on 0.7% and 2.5% agar with and without 5 μm of oryzalin. Scale bar = 50 μm. **(C)** Bursting index (violin plot) and *p*-values of Wilcoxon–Mann–Whitney tests in *pPDF1*::*mCit-MBD* and *fer-4 pPDF1*::*mCit-MBD* hypocotyls grown on 0.7% or 2.5% agar, with and without 5 μm of oryzalin. **(D)** In the WT (bottom), cells resist mechanical stress (red arrows) by 2 independent pathways (microtubule-dependent cellulose synthesis and FER-dependent wall reinforcement). In absence of both FER and microtubules (top), cells deform like passive matter and ultimately burst. All underlying data can be found in S5 Data. FER, FERONIA; WT, wild type.

Our ablation results may seem at odds with the fact that *fer-4* and *bot1-7* belong to the same cluster when analyzing pavement cell circularity (see Fig 1D). We thus revisited our quantification of pavement cell shape to identify other shape descriptors amenable to discriminate *bot1-7* and *fer-4*. We focused on lobe size in pavement cells. In the katanin mutant *bot1-7*, which displayed a higher circularity than the WT (see Fig 1), the average basal lobe width was 3% smaller than that of the WT (*p* < 10^−3^; *n*_WS-4_ = 294; *n*_*bot1-7*_ = 252; Fig 5A). Although this is a rather small difference, this is consistent with a reduced microtubule response to stress in the katanin mutant. By contrast, the average basal lobe width in *fer-4* was 12% larger than that of the WT (*p* < 10^−3^; *n*_*Col-0*_ = 428; *n*_*fer-4*_ = 321; Fig 5A). Similar trends in basal lobe width were observed when seedlings were grown on 2.5% agar, albeit with lower values, also consistent with a reduced microtubule response to mechanical stress in such conditions (S10 Fig). Thus, both *fer-4* and *bot1-7* pavement cells exhibit higher circularity than the WT through different geometries. Pavement cell shape may thus reflect different responses to stress: Reduced microtubule dynamics in *bot1-7* would generate smaller lobes, whereas increased stress levels in *fer-4* would lead to hyperaligned cortical microtubules and larger lobes. Such hyperaligned cortical microtubules can be observed in *fer-4* pavement cells when seedlings are grown on 0.7% agar (S6C Fig). Alternatively, and nonexclusively, the presence of dead cells in *fer-4* may affect the stress pattern, and thus the cortical microtubule response, further increasing the circularity of pavement cells.

Treatment with the microtubule depolymerizing drug oryzalin has previously been shown to induce cell bursting events in the largest cells at the shoot apical meristem [23], mimicking the bursting cell phenotype we observe in hypocotyls and cotyledons in *fer-4*. If both pathways are truly independent, then one should expect additive behaviors. To test this prediction, we observed hypocotyl growth when both pathways are down, by applying oryzalin on *fer-4* mutants. On 0.7% agar, bursting cells appeared in both oryzalin-treated hypocotyls and control ones in *fer-4*. The bursting index increased by 192% in oryzalin-treated *fer-4* hypocotyls, consistent with an additive role of both pathways in wall integrity (*p*_*fer-4*,0.7%agar_ < 10^−3^; n_*fer-4*,DMSO,0.7%agar_ = 10; n_*fer-4*,oryzalin,0.7%agar_ = 11, Fig 5B and 5C). To relate this phenotype to stress levels, we performed the same experiment on 2.5% agar and found a reduction in the bursting cells in all conditions (by 71% on DMSO; *p*_*fer-4*,DMSO_ = 0.002; n_*fer-4*,DMSO,0.7%agar_ = 10; n_*fer-4*,DMSO,2.5%agar_ = 9; by 67% on oryzalin; *p*_*fer-4*,oryzalin_ < 10^−3^; n_*fer-4*,oryzalin,0.7%agar_ = 11; n_*fer-4*,oryzalin,2.5%agar_ = 10; Fig 5B and 5C). This further confirms that FER and cortical microtubules independently contribute to the response to stress.

## Discussion

From a reverse genetic screen, we show that FER plays a primary role in the mechanical integrity of the shoot (using both hypocotyl and cotyledons as model systems). We then find that FER prevents turgor-dependent cell bursting in the shoot. We also find that the microtubule response to stress does not require FER. This does not exclude the possibility that FER may modulate the microtubule response to stress, indirectly. Finally, we show that shutting down both FER and microtubule pathways has an additive effect on turgor-dependent cell bursting. This provides a scenario in which the mechanical feedback in the shoot that is required to maintain mechanical integrity, involves 2 largely independent modules, cortical microtubules and FER (Fig 5D).

This result is consistent with recent findings showing how pectin and cellulose deposition are controlled with largely disconnected networks: Pectins are important for the initiation of pavement cell formation, and the deposition of cellulose microfibril rather amplifies a preexisting mechanical pattern [24]. Similarly, mechanical polarities in growing hypocotyl cells precedes the cellulose-derived mechanical anisotropy in hypocotyl cells [16].

We also provide evidence that the *fer* mutant can be largely rescued through changes in the mechanical environment of the plant despite its central and somewhat pleiotropic role in plant development. This echoes recent findings where essential regulators are found optional, when challenged with different mechanical environments. This is the case for instance for key regulators of pectin synthesis, where decreasing tensile stress can rescue cell–cell adhesion defects in the *quasimodo* mutants [13]. This was also shown for the katanin mutant, where increasing tensile stress levels with isoxaben treatment can generate WT-like cortical microtubule arrays [29]. More recently, this was even extended to signaling in the context of apical hook formation: *arf7 arf19* auxin transduction mutant seedlings exhibit a WT phenotype when mechanically constrained [45].

If the microtubule response to stress does not depend on FER, what could be the most relevant mechanotransduction pathway? As shown with optical tweezers, pulling on microtubules promote their polymerization in vitro [46,47]. Cortical microtubules also align with predicted maximal tension when protoplasts are confined in rectangular microwells and pressurized by hypoosmotic conditions [48]. These data, together with the FER-independent microtubule response to stress, further support the hypothesis that microtubules may be their own mechanosensor [49].

Conversely, microtubule (and cellulose microfibril) alignment likely results from a combination of several cues, beyond tensile stress. Cell geometry can affect microtubule behavior, independent of cortical tension. In particular, due to their high persistence length, microtubules tend to become longitudinal in vitro [50] or in depressurized protoplasts [51]. Furthermore, cell edge factors can affect cortical microtubule behavior, leading to cell-scale aligned arrays [52–54]. Although microtubule, FER, stress, and cell geometry can be uncoupled in experiments, their interplay may provide synergies in vivo. In particular, the deposition of matrix material in the wall depends on exocytosis, which is also promoted by membrane tension. Conversely, affecting matrix deposition may weaken the wall and increase the tensile stress levels. Consistently, microtubule arrays exhibit an enhanced anisotropy when the cell edge GTPase Rab-A5c dependent trafficking is affected in roots [54].

All living organisms constantly sense and respond to mechanical stress. Interestingly, so far, all known mechanosensors at the extracellular matrix (e.g., integrins) also display ligand binding activity. FER may belong to this category as well, as FER also acts as a RALF peptide receptor. Whether this dual role provides synergistic activity for signaling remains to be investigated.

By impairing FER and microtubules, we reveal how plant cells behave when they become unable to respond to stress: They switch to a more passive mode, like a balloon or a soap bubble, and because of turgor pressure, swell and ultimately die by bursting. Revisiting living systems with the lens of active matter physics is thus particularly suited to understand how cells manage to resist to stress, notably in their response to a fluctuating environment.

## Materials and methods

### Plant material and growth conditions

All plants were in the *Columbia-0* (*Col-0*) ecotype, except the *bot1-7* mutant, which was in the *Wassilewskija-4* (*WS-4*) ecotype (S1 Table).

Seeds were surface sterilized and individually sown on Murashige and Skoog medium (MS medium, Duchefa, Haarlem, the Netherlands) or *Arabidopsis* medium (custom-made Duchefa “*Arabidopsis*” medium (DU0742.0025, Duchefa Biochemie, The Netherlands) with different agar concentrations (see S2 Table for detailed description of the different media used). For drug treatments, media were supplemented with isoxaben (Sigma, Germany) or oryzalin (Chem Service, USA) from stock solutions in dimethyl sulfoxide (DMSO, Sigma). All plants were placed 2 days in the dark at 4°C then transferred in a 20°C long days growth chamber. When seedlings were maintained in the dark, petri dishes were covered with aluminum foil in the 20°C long days growth chamber.

### Image acquisition

Samples were imaged with either a SP8 confocal microscope (Leica Microsystems, Germany) equipped with a 25× long-distance water objective (NA = 0.95), an Epson Perfection 2400 scanner, or a Leica MZ12 microscope (as specified in S2 Table). Samples were stained for 10 minutes with a 1/10 propidium iodide solution (Sigma; PI stains wall pectins and thus marks cell contours). Ablations (Fig 4) were performed manually with a fine needle (Minutien pin, 0.15-mm rod diameter, 0.02-mm tip width, RS-6083–15, Roboz Surgical Instrument, USA) as described in [13]. In all confocal microscopy images, 0.5-μm thick optical slices were acquired.

For every experiment, 3 biological replicates or more were obtained. *Col-0* or *WS-4* seedlings were included as controls in all experiments and replicates. This also explains why the sample size of *Col-0* images used in Figs 1 and 2 is bigger than for the other genotypes. Note that some of the *Col-0* cotyledon data were previously used in our methodological article [55], as templates to introduce the SurfCut ImageJ tool (see below in Image analysis) for cell contour extraction.

### Image analysis

Pavement cell shape were obtained by first processing confocal images with MorphoGraphX (http://www.mpipz.mpg.de/MorphoGraphX) [56] to obtain cell contours in a 2.5D epidermal surface (Figs 1 and 5A) or SurfCut (https://github.com/sverger/SurfCut) [55] to extract the flattened cell contours (Fig 3E). The cell contour images were then processed with PaCeQuant [27], an ImageJ plug-in quantifying up to 27 shape descriptors of pavement cells. Hypocotyl length (Fig 2) was measured manually with ImageJ (https://fiji.sc/). Cell burst area (Figs 3, 5C and 5D) was measured manually with ImageJ after extracting the flattened cell contours with SurfCut (https://github.com/sverger/SurfCut) [55]. Cotyledon area (Fig 3) was measured manually with ImageJ. Microtubule organization (Fig 4) was quantified with FibrilTool [57] after flattening the images with SurfCut and denoising them (ROF Denoise, Theta = 25) in ImageJ, as performed in [13]. After image analyses, the brightness and contrast of all images presented in this study were enhanced to help visualization.

### Statistical analysis

Statistical analyses were performed with R software (https://www.R-project.org). The sample size is indicated in the main text. For pavement cell shapes (Figs 1 and 5A), we used PaCeQuantAna, the R script that accompanies the PaCeQuant analysis [27], and the FactoMineR and factoextra R libraries for the principal component analysis [58]. Violin plots were shown with the corresponding *p*-value of Kruskal–Wallis tests. For hypocotyl length (Fig 2), the control distribution of hypocotyl length was standardized and the same parameters (μ_ctrl_, ơ_ctrl_) used to shift the isoxaben distribution similarly (S2 Fig). A Wilcoxon–Mann–Whitney test was then performed on the shifted distributions of the mutant and of the WT for each genotype. For the orientation of cortical microtubules, a Kolmogorov–Smirnov test was performed to compare the angle distributions to a uniform distribution between 0° and 90°. All other quantitative measures were compared using Wilcoxon–Mann–Whitney tests. As a Wilcoxon–Mann–Whitney test can be directed or not, the *p*-value shown in all experiments with a Wilcoxon–Mann–Whitney test was that of a nondirected test for nonsignificant *p*-value (to ensure that neither distribution was higher than the other) and that of a directed test in the significant direction for a significant *p*-value. Differences with *p*-values that were under 1% were considered significant, and those between the commonly used thresholds of 5% and 1% were considered as tendencies. All ± values referred to the standard deviation of the distribution.

## Supporting information

S1 Table Accessions. (DOCX)

S2 Table Growth and imaging conditions. (DOCX)

S1 Fig Analysis of pavement cell shapes. **(A)** Pipeline for the extraction and analysis of pavement cell shape. Epidermal signal was first extracted with MorphoGrahX, then segmented with the PaCeQuant ImageJ plug-in, which extracted 27 pavement cell shape descriptors (parameters). Finally, the results were processed by PaCeQuantAna (the PaCeQuant R script). **(B)** Main shape descriptors of pavement cells. Nonlobe area (in μm^2^) stands for the area of a cell without the lobes. Circularity is the area of a cell divided by its square perimeter, with a normalization to have a maximal circularity of 1 for a circle. Solidity is the area of a cell divided by the area of its convex hull (convex polygon with the smallest area including the whole cell). Average basal lobe width (μm) stands for the average length of the bases of a cell’s lobes. **(C)** Nonlobe area (violin plots) of pavement cells and *p*-values of Dunn tests in the WT (in blue, *Col-0* and *WS-4*), in microtubule associated mutants (in orange, *nek6-1*, *spr2-2*, *tua3*, *tua4*, *tua5*, and *bot1-7*) and in receptor-like kinase mutants (in pink, *tfr1-1*, *cvy1-1*, *fer-4*, *herk1-1*, *herk2-1*, *the1-4*, *the1-6*, *wak1-1*, *wak2-1*, *wak3-1*, *wak4-1*, and *mik2-1*). **(D)** Solidity (violin plots) of pavement cells and *p*-values of Dunn tests in the WT (in blue, *Col-0*, *WS-4*), in microtubule associated mutants (in orange, *nek6-1*, *spr2-2*, *tua3*, *tua4*, *tua5*, and *bot1-7*) and in receptor-like kinase mutants (in pink, *tfr1-1*, *cvy1-1*, *fer-4*, *herk1-1*, *herk2-1*, *the1-4*, *the1-6*, *wak1-1*, *wak2-1*, *wak3-1*, *wak4-1*, and *mik2-1*). All underlying data can be found in S1 Data. WT, wild type.
(JPG)

S2 Fig Definition of the hypocotyl length index. The continuous black and red lines represent the distribution of the actual measurement of hypocotyl lengths for the treated (red) and control (black) samples. In this example, the values are between 0 and 2 cm, and the average values for the treated sample are lower. However, when comparing different genotypes, the average length of the untreated (control) mutant samples could already be lower or higher than the control WT samples, thus hampering a direct comparison of the effect of the treatment on the mutant. In order to allow statistical comparison between the samples we normalized the distribution of hypocotyl lengths in the presence of isoxaben according to the distribution of hypocotyl lengths in control conditions, following the standard score method. To do so, the parameters of the control distribution (DMSO, mean: μ_ctrl_, standard deviation: ơ_ctrl_) are shifted around 0 (dashed black line) and are used to shift the isoxaben distribution to the same extent (dashed red line). With this method, the control samples for all genotypes have a value around 0, which then allows to reveal the effect of the treatment on the different mutants by comparing the means of the normalized treated samples (the hypocotyl length index; Fig 2B). To further ease the comparison, the differences between WT and mutants are then plotted as the hypocotyl length index deviation from the WT, which are the values obtained when subtracting the mutant treated normalized mean to the WT treated normalized mean. This generates a negative value in the hypocotyl length index for mutants being more sensitive to the treatment and a positive value for mutants less sensitive to the treatment (Fig 2C). WT, wild type.
(PNG)

S3 Fig *fer-2* phenotypes. **(A)** Representative images of Col-0 and *fer-2* pavement cells. Samples were PI stained and cell contours were extracted with MorphoGraphX and projected in 2D. Scale = 100 μm. **(B)** Representative images of Col-0 and *fer-2* etiolated seedlings grown with or without 1 nM isoxaben. Scale = 1 cm. **(C)** Representative confocal images of *Col-0* and *fer-2* pavement cells, from seedlings grown on a medium containing 0.7% or 2.5% agar at t = 4 DAG and t = 12 DAG (propidium iodide staining). Scale = 100 μm. **(D)** Representative images of *Col-0* and *fer-2* cotyledons, grown on a medium containing 0.7% or 2.5% agar at t = 4 DAG, t = 8 DAG and t = 12 DAG. Scale = 1 mm. **(E)** Representative confocal images of Col-0 and *fer-2* hypocotyls grown on 0.7% and 2.5% agar with and without 5 μm of oryzalin. All underlying data can be found in Supporting information folder. Scale bar = 50 μm. PI, propidium iodide.
(JPG)

S4 Fig Swelling pavement cells in *fer-4* on 0.7% agar. **(A, B)** Representative images of *Col-0 (A) and fer-4 (B)* pavement cells, from seedlings grown on a medium containing 0.7% agar at t = 4 DAG. Samples were PI stained and cell contours were extracted with SurfCut and projected in 2D. Orthogonal projections were also extracted with Fiji. Note the presence of curvy outer walls in some *fer-4* cells. All underlying data can be found in Supporting information folder. Scale = 100 μm. PI, propidium iodide.
(JPG)

S5 Fig Bursting cells in *fer-4* upon switch from 2.5% to 0.35% agar conditions. **(A, B)** Representative confocal images of *fer-4* pavement cells, from seedlings grown on a medium containing 2.5% agar for 4 days, transferred on a medium containing 0.35% agar, stained with PI and imaged in water immediately after the transfer to a new medium (t0) and 1 to 16 hours later. Cell contours were extracted with SurfCut and projected in 2D. Orthogonal projections were also extracted with Fiji, focusing on the cell indicated with red arrows. See also S1 Movie. Scale = 100 μm.
(JPG)

S6 Fig Cortical microtubule behavior in *fer-4* grown on 0.7% agar medium. Representative confocal images of *pPDF1*::*mCit-MBD* **(A)** and *fer-4 pPDF1*::*mCit-MBD* **(B)** pavement cells from seedlings grown on a medium containing 0.7% agar. Quantification of the anisotropy of cortical microtubule arrays in both lines **(C)**. Note the presence of circumferential cortical microtubules around dead cells, matching the predicted maximal tensile stress direction. Representative confocal images of *pPDF1*::*mCit-MBD* **(D, E)** and *fer-4 pPDF1*::*mCit-MBD* **(F, G)** pavement cells from seedlings grown on a medium containing 0.7% agar, immediately after ablation (t0, D, F) and 7 hours later (t7h, E, G). Note the presence of many dead cells and the strong alignment of cortical microtubules at t0 in *fer-4*. All underlying data can be found in Supporting information folder. Scale = 50 μm.
(JPG)

S7 Fig Ablations (full kinetics) on 2.5% agar medium (example 1). **(A)** Representative confocal images of *pPDF1*::*mCit-MBD* (top) and *fer-4 pPDF1*::*mCit-MBD* (bottom) pavement cells, from seedlings grown on a medium containing 2.5% agar, immediately after an ablation (t0) and 1 to 7 hours later. Scale = 50 μm. **(B)** Orientation to the ablation (violin plot) of cortical microtubule arrays and *p*-values of Wilcoxon–Mann–Whitney tests in cells surrounding the ablation site in *pPDF1*::*mCit-MBD* and *fer-4 pPDF1*::*mCit-MBD* pavement cells, immediately after an ablation (t0) and 7 hours later (t7h) (see Fig 4). All underlying data can be found in Supporting information folder.
(JPG)

S8 Fig Ablations (full kinetics) on 2.5% agar medium (example 2). **(A)** Representative confocal images of *pPDF1*::*mCit-MBD* (A) and *fer-4 pPDF1*::*mCit-MBD* **(B)** pavement cells, from seedlings grown on a medium containing 2.5% agar, immediately after an ablation (t0) and 1 to 7 hours later. Scale = 50 μm.
(JPG)

S9 Fig Microtubule response after ablation using the GFP-tubulin reporter. Representative confocal images of *p35S*::*GFP-TUB* (top) and *fer-4 p35S*::*GFP-TUB* (bottom) pavement cells from seedlings grown on a medium containing 2.5% agar, immediately after an ablation (t0) and 7 hours later (t7h). Scale = 50 μm.
(JPG)

S10 Fig Basal lobe width according to genotype and growth conditions. Basal lobe width (violin plot) of pavement cells and *p*-values of Dunn tests for the WT (*Col-0*, *WS-4*), katanin mutant (*bot1-7*), and *fer-4*. Seedlings were grown on 0.8% or 2.5% agar. Data from Fig 5A (0.8% agar) are reproduced here to allow a full comparison of the results. All underlying data can be found in S7 Data. WT, wild type.
(PNG)

S1 Movie Bursting cells in *fer-4* upon switch from 2.5% to 0.35% agar conditions. Animation of representative confocal images of *fer-4* pavement cells, from seedlings grown on a medium containing 2.5% agar for 4 days, transferred on a medium containing 0.35% agar, stained with PI and imaged in water immediately after the transfer to a new medium (t0) and 1 to 16 hours later. Cell contours were extracted with SurfCut and projected in 2D (same images as in S5 Fig) then aligned with the stackreg (Pyramid Registration) plug-in in Fiji. PI, propidium iodide.
(AVI)

S1 Data Data related to Fig 1 (panels B, C, and D), Fig 5 (panel A), and S1 Fig (panels C and D).
(XLSX)

S2 Data Data related to Fig 2 (panels B and C).
(XLSX)

S3 Data Data related to Fig 3 (panels B, D, and F).
(XLSX)

S4 Data Data related to Fig 4 (panels E–I) and S7 Fig (panel B).
(XLSX)

S5 Data Data related to Fig 5 (panel C).
(XLSX)

S6 Data Data related to S6 Fig (panel C).
(XLSX)

S7 Data Data related to S10 Fig.
(XLSX)

## Abbreviations

CrRLK: *Catharanthus roseus* Receptor-Like Kinase
*cvy1*: *curvy1*
FER: FERONIA
herk1: *hercules receptor kinase 1*
*mik2-1*: *mdis1-interacting receptor-like kinase2*
PI: propidium iodide
ROS: reactive oxygen species
*tfr1*: *theseus1/feronia-related1*
THE1: THESEUS1
*wak*: *wall-associated kinase*
WT: wild type
