# Supplementary material for: FERONIA and microtubules independently contribute to mechanical integrity in the Arabidopsis shoot
Source: PLoS Biol. 2021 Nov 12;19(11):e3001454. doi: 10.1371/journal.pbio.3001454 (PMC8612563; doi:10.1371/journal.pbio.3001454)
Supplement: S1 Table — (DOCX) [file pbio.3001454.s001.docx]

**Supplementary Table 1.** Accessions

| **ID** | **Line** | **Full gene name** | **AGI** | **Ontology** | **Ecotype** | **Reference** |
| --- | --- | --- | --- | --- | --- | --- |
| *nek6-1* | SALK_152782 | *NIMA (NEVER IN MITOSIS, GENE A)-RELATED 6* | AT3G44200 | Tubulin kinase | Col-0 | Motose *et al.,* 2008. Plant J Cell Mol Biol 54(5):829–844. |
| *spr2-2* | EMS CS6549 | *SPIRAL2* | AT4G27060 | MAP | Col-0 | Shoji *et al.,* 2004. Plant Physiol 136(4):3933–3944. |
| *tua3(D205N)* | EMS CS68877 | *TUBULIN ALPHA-3* | AT5G19770 | Tubulin | Col-0 | Ishida et al., 2007. Proc Natl Acad Sci U S A 104(20):8544–8549. |
| *tua4(S178D)* | EMS CS68881 | *TUBULIN ALPHA-4* | AT1G04820 | Tubulin | Col-0 | Ishida et al., 2007. Proc Natl Acad Sci U S A 104(20):8544–8549. |
| *tua5(D251N)* | EMS CS68884 | *TUBULIN ALPHA-5* | AT5G19780 | Tubulin | Col-0 | Ishida et al., 2007. Proc Natl Acad Sci U S A 104(20):8544–8549.. |
| *tfr1-1* | GABI 649_E11 | THESEUS1/FERONIA-RELATED1 | AT5G24010 | CrRLK | Col-0 | This study, provided by H. Höfte |
| *cvy1-1* | SALK_018797 | *CURVY1* | AT2G39360 | CrRLK | Col-0 | Gachomo *et al.,* 2014. BMC Plant Biol 14:221. |
| *fer-4* | GABI 106_A06 | *FERONIA* | AT3G51550 | CrRLK | Col-0 | Duan et al., 2010. Proc Natl Acad Sci U S A 107(41):17821–17826. |
| *fer-2* | TAG insert | *FERONIA* | AT3G51550 | CrRLK | Col-0 | Deslauriers and Larsen, 2010. Molecular Plant 3, 626–640 |
| *herk1-1* | SALK_008043 | *HERCULES RECEPTOR KINASE 1* | AT3G46290 | CrRLK | Col-0 | Guo et al., 2009. Proc Natl Acad Sci U S A 106(18):7648–7653. |
| *herk2-1* | SALK_105055 | *HERCULES RECEPTOR KINASE 2* | AT1G30570 | CrRLK | Col-0 | Guo et al., 2009. Proc Natl Acad Sci U S A 106(18):7648–7653. |
| *the1-6* | EMS (sup ctl1-2) | *THESEUS1* | AT5G54380 | CrRLK | Col-0 | Merz *et al.,* 2017. J Exp Bot 68(16):4583–4593. |
| *wak1-1* | SALK_107175 | *CELL WALL-ASSOCIATED KINASE 1* | AT1G21250 | WAK | Col-0 | He *et al.,* 1996. J Biol Chem 271(33):19789–19793. |
| *wak2-1* | SAIL_286_E03 | *CELL WALL-ASSOCIATED KINASE 2* | AT1G21270 | WAK | Col-0 | He *et al.,* 1996. J Biol Chem 271(33):19789–19793. |
| *wak3-1* | SALK_071999 | *CELL WALL-ASSOCIATED KINASE 3* | AT1G21240 | WAK | Col-0 | He et al., 1999. Plant Mol Biol 39(6):1189–1196. |
| *wak4-1* | SAIL_1156_F08 | *CELL WALL-ASSOCIATED KINASE 4* | AT1G21210 | WAK | Col-0 | He et al., 1999. Plant Mol Biol 39(6):1189–1196. |
| *mik2-1* | SALK_061769 | *MDIS1-INTERACTING RECEPTOR LIKE KINASE2* | AT4G08850 | LRR-RLK | Col-0 | Wang *et al.,* 2016. Nature 531(7593):241–244. |
| *bot1-7* | deletion of 19 bp | *KATANIN 1* | AT1G80350 | Katanin | WS-4 | Bichet *et al.,* 2001. Plant J 25(2):137–148. |
| *pPDF1::mCit-MBD* | Translational fusion protein | | | Microtubule marker | Col-0 | Armezzani *et al.,* 2018. Development 145. |
| *fer-4 pPDF1::mCit-MBD* | Translational fusion protein | | | Microtubule marker | Col-0 | Cross, this study |
| *p35S::GFP-TUB* | Translational fusion protein | | | Microtubule marker | Col-0 | Lin et al., 2018) BioRxiv. https://doi.org/10.1101/269647 |
| *fer-4 p35S::GFP-TUB* | Translational fusion protein | | | Microtubule marker | Col-0 | Lin et al., 2018) BioRxiv. https://doi.org/10.1101/269647 |

Abbreviations: MAP: Microtubule Associated Protein, CrRLK: Catharanthus roseus Receptor-Like Kinase, WAK: Wall Associated Kinase, LRR-RLK: Leucin Rich Repeat Receptor-Like Kinase
