# Supplementary material for: FERONIA and microtubules independently contribute to mechanical integrity in the Arabidopsis shoot
Source: PLoS Biol. 2021 Nov 12;19(11):e3001454. doi: 10.1371/journal.pbio.3001454 (PMC8612563; doi:10.1371/journal.pbio.3001454)
Supplement: S2 Table — (DOCX) [file pbio.3001454.s002.docx]

**Supplementary Table 2.** Growth and imaging conditions

| **Figure** | **Panel** | | **Medium** | **Growth conditions** | **Imaging** |
| --- | --- | --- | --- | --- | --- |
| Figure 1 | A-D | | Medium A | Condition 1 | Imaging a |
| Figure 2 | A-C | | Medium B | Condition 2 | Imaging b |
| Figure 3 | A-B | | Medium C | Condition 2 | Imaging c |
| Figure 3 | C-D | | Medium D | Condition 3 | Imaging a |
| Figure 3 | E-F | | Medium D | Condition 3 | Imaging f |
| Figure 4 | A-I | | Medium D | Condition 1 | Imaging d |
| Figure 5 | A | | Medium A | Condition 1 | Imaging a |
| Figure 5 | B-C | | Medium E | Condition 4 | Imaging e |
| **Growth medium** | | | | | |
| Medium A | | MS medium with 0.8% agar, 1% sucrose, and no vitamin | | | |
| Medium B | | MS medium with 0.8% agar, no sucrose, and no vitamin supplemented with 1 nM of isoxaben diluted in DMSO, or the same volume of DMSO (control) | | | |
| Medium C | | MS medium with 0.8% or 2,5% agar, no sucrose, and no vitamin supplemented with 1 nM of isoxaben diluted in DMSO, or the same volume of DMSO | | | |
| Medium D | | Arabidopsis medium with 0,7% or 2,5% agar and no vitamin | | | |
| Medium E | | Arabidopsis medium with 0,7% or 2,5% agar, no vitamin, and supplemented with 5 µM of oryzalin diluted in DMSO, or the same volume of DMSO | | | |
| **Growth conditions** | | | | | |
| Condition 1 | | 8 hours light, 3 days darkness, 3 days light | | | |
| Condition 2 | | 8 hours light, 4 days darkness | | | |
| Condition 3 | | 4 days light, 8 days light or 12 days light | | | |
| Condition 4 | | 5 days light | | | |
| **Imaging**  In all confocal microscopy image acquisition, optical sections were 0.5 µm thick. | | | | | |
| Imaging a | | Samples were transferred to new medium plates, stained with a PI solution (dilution 1/10), and rinsed twice. Cotyledons were manually set in flat position with forceps under a binocular stereoscopic microscope and imaged with a long distance 25x objective (Leica SP8). | | | |
| Imaging b | | The petri dish lid was removed and the petri dish was scanned with an office scanner. | | | |
| Imaging c | | Samples were stained with PI (1/100 dilution), rinsed once, mounted in water between slides and coverslips, and imaged with a long distance 25x objective (Leica SP8). | | | |
| Imaging d | | Samples were transferred to new medium plates. Cotyledons were manually set in flat position with forceps under a binocular stereoscopic microscope, and imaged with a 25x long distance objective (Leica SP8). | | | |
| Imaging e | | Samples were stained with PI (1/100 dilution), rinsed once, mounted in medium and water between slides and coverslips, and imaged with a long distance 25x objective (Leica SP8). | | | |
| Imaging f | | Dissected cotyledons were mounted in water between slides and coverslips and imaged with a binocular stereomicroscope (Leica MZ12). | | | |
