# Supplementary figures and images for: FERONIA and microtubules independently contribute to mechanical integrity in the Arabidopsis shoot
Source: PLoS Biol. 2021 Nov 12;19(11):e3001454. doi: 10.1371/journal.pbio.3001454 (PMC8612563; doi:10.1371/journal.pbio.3001454)

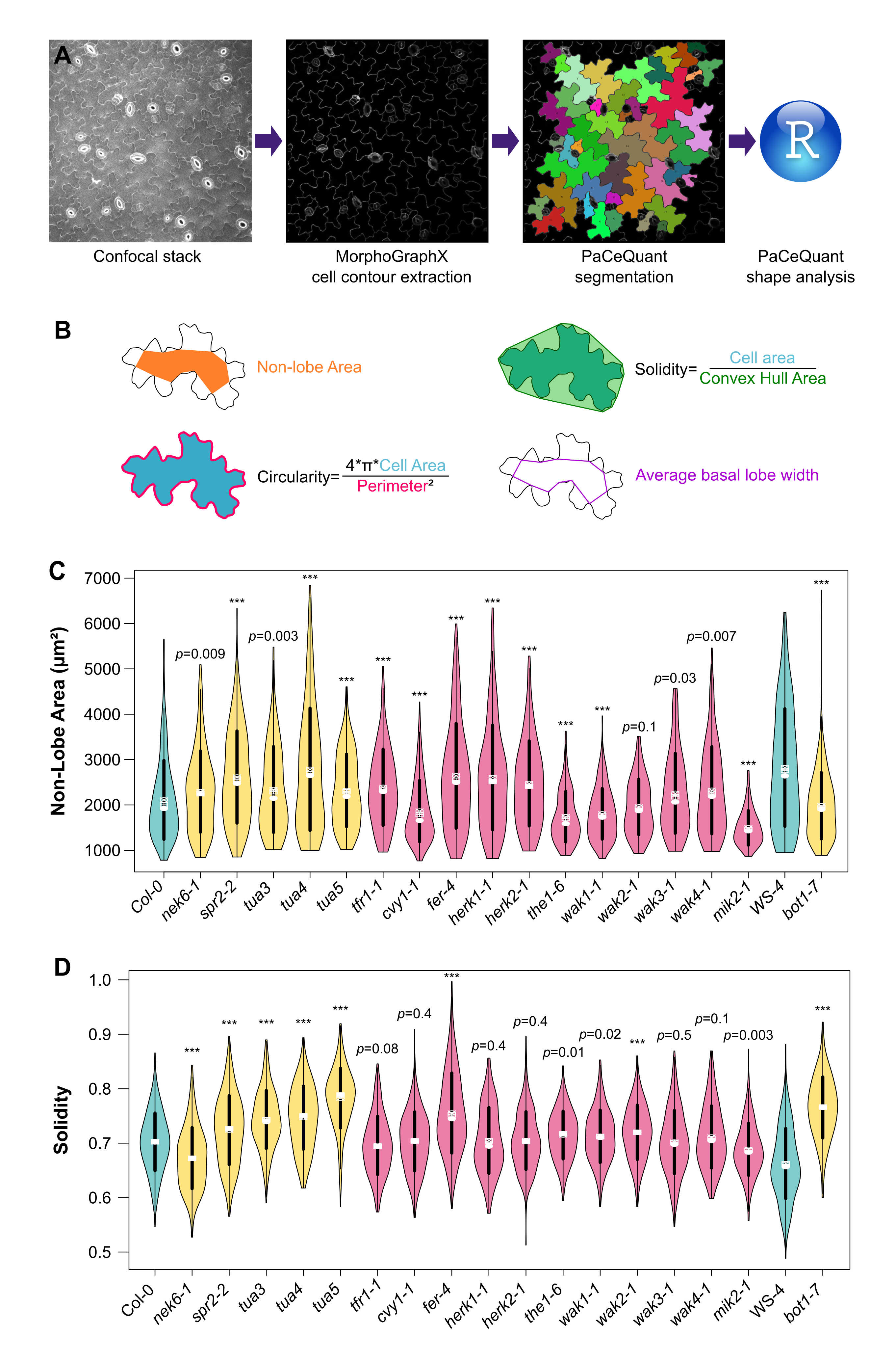

Supplement: S1 Fig — (A) Pipeline for the extraction and analysis of pavement cell shape. Epidermal signal was first extracted with MorphoGrahX, then segmented with the PaCeQuant ImageJ plug-in, which extracted 27 pavement cell shape descriptors (parameters). Finally, the results were processed by PaCeQuantAna (the PaCeQuant R script). (B) Main shape descriptors of pavement cells. Nonlobe area (in μm2) stands for the area of a cell without the lobes. Circularity is the area of a cell divided by its square perimeter, with a normalization to have a maximal circularity of 1 for a circle. Solidity is the area of a cell divided by the area of its convex hull (convex polygon with the smallest area including the whole cell). Average basal lobe width (μm) stands for the average length of the bases of a cell’s lobes. (C) Nonlobe area (violin plots) of pavement cells and p-values of Dunn tests in the WT (in blue, Col-0 and WS-4), in microtubule associated mutants (in orange, nek6-1, spr2-2, tua3, tua4, tua5, and bot1-7) and in receptor-like kinase mutants (in pink, tfr1-1, cvy1-1, fer-4, herk1-1, herk2-1, the1-4, the1-6, wak1-1, wak2-1, wak3-1, wak4-1, and mik2-1). (D) Solidity (violin plots) of pavement cells and p-values of Dunn tests in the WT (in blue, Col-0, WS-4), in microtubule associated mutants (in orange, nek6-1, spr2-2, tua3, tua4, tua5, and bot1-7) and in receptor-like kinase mutants (in pink, tfr1-1, cvy1-1, fer-4, herk1-1, herk2-1, the1-4, the1-6, wak1-1, wak2-1, wak3-1, wak4-1, and mik2-1). All underlying data can be found in S1 Data. WT, wild type. (JPG) [file pbio.3001454.s003.jpg]

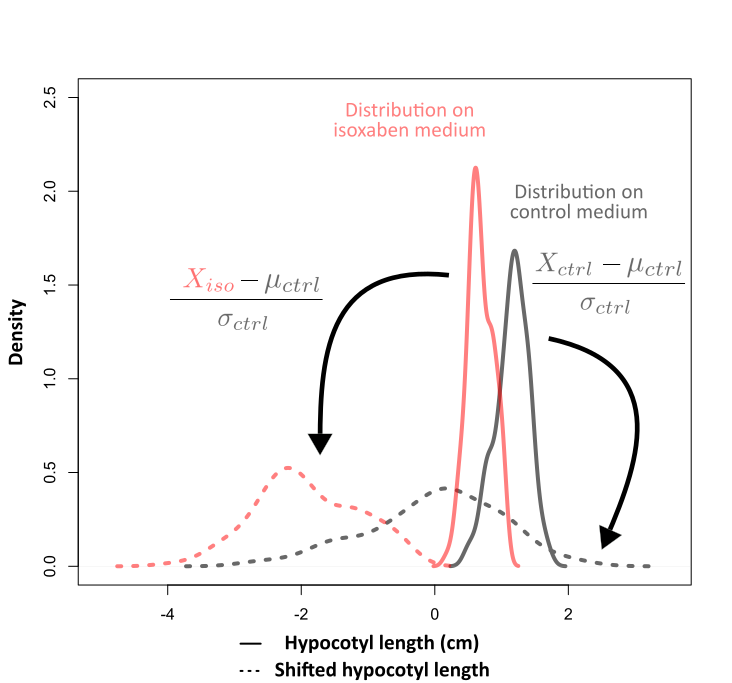

Supplement: S2 Fig — The continuous black and red lines represent the distribution of the actual measurement of hypocotyl lengths for the treated (red) and control (black) samples. In this example, the values are between 0 and 2 cm, and the average values for the treated sample are lower. However, when comparing different genotypes, the average length of the untreated (control) mutant samples could already be lower or higher than the control WT samples, thus hampering a direct comparison of the effect of the treatment on the mutant. In order to allow statistical comparison between the samples we normalized the distribution of hypocotyl lengths in the presence of isoxaben according to the distribution of hypocotyl lengths in control conditions, following the standard score method. To do so, the parameters of the control distribution (DMSO, mean: μctrl, standard deviation: ơctrl) are shifted around 0 (dashed black line) and are used to shift the isoxaben distribution to the same extent (dashed red line). With this method, the control samples for all genotypes have a value around 0, which then allows to reveal the effect of the treatment on the different mutants by comparing the means of the normalized treated samples (the hypocotyl length index; Fig 2B). To further ease the comparison, the differences between WT and mutants are then plotted as the hypocotyl length index deviation from the WT, which are the values obtained when subtracting the mutant treated normalized mean to the WT treated normalized mean. This generates a negative value in the hypocotyl length index for mutants being more sensitive to the treatment and a positive value for mutants less sensitive to the treatment (Fig 2C). WT, wild type. (PNG) [file pbio.3001454.s004.png]

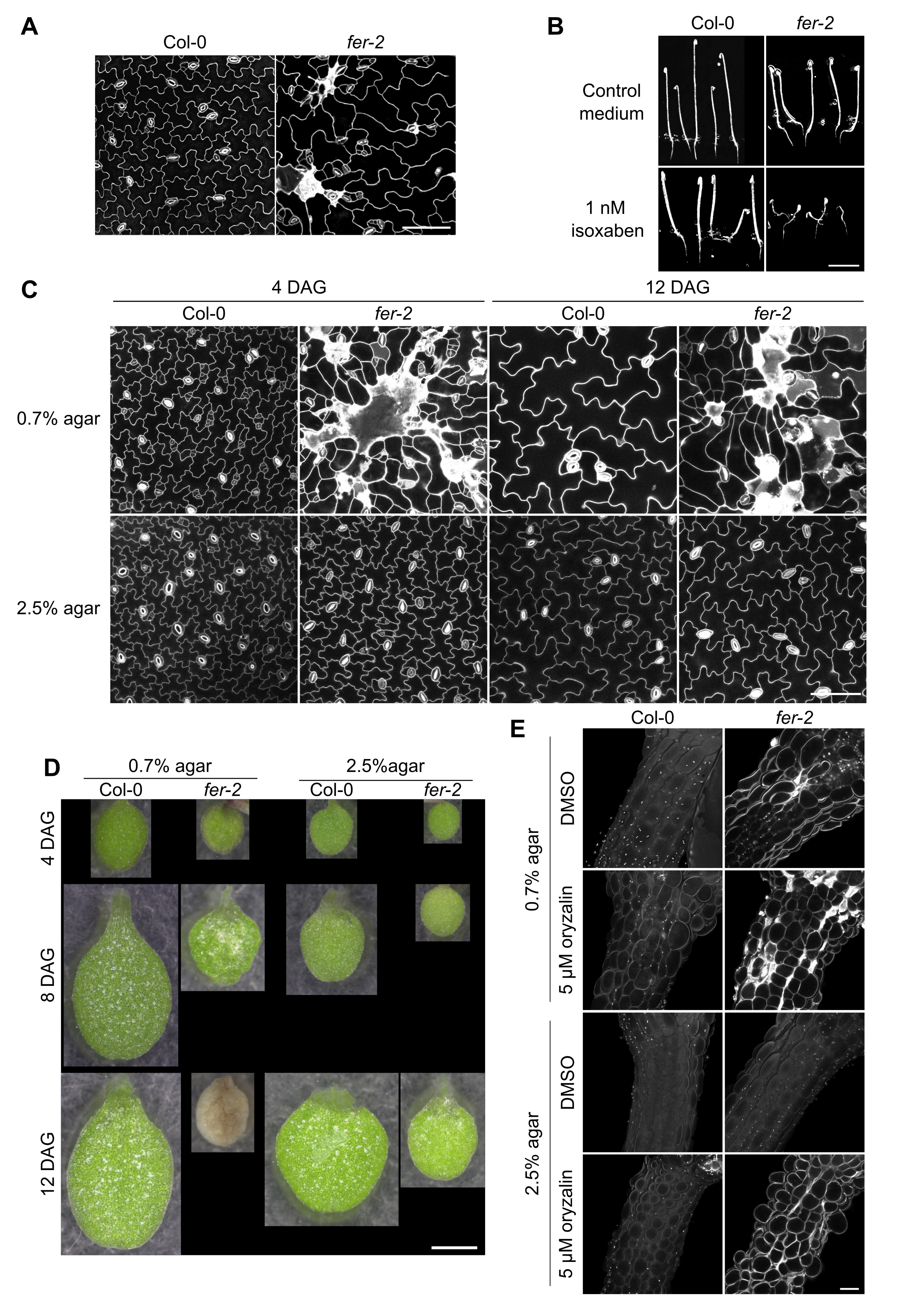

Supplement: S3 Fig — (A) Representative images of Col-0 and fer-2 pavement cells. Samples were PI stained and cell contours were extracted with MorphoGraphX and projected in 2D. Scale = 100 μm. (B) Representative images of Col-0 and fer-2 etiolated seedlings grown with or without 1 nM isoxaben. Scale = 1 cm. (C) Representative confocal images of Col-0 and fer-2 pavement cells, from seedlings grown on a medium containing 0.7% or 2.5% agar at t = 4 DAG and t = 12 DAG (propidium iodide staining). Scale = 100 μm. (D) Representative images of Col-0 and fer-2 cotyledons, grown on a medium containing 0.7% or 2.5% agar at t = 4 DAG, t = 8 DAG and t = 12 DAG. Scale = 1 mm. (E) Representative confocal images of Col-0 and fer-2 hypocotyls grown on 0.7% and 2.5% agar with and without 5 μm of oryzalin. All underlying data can be found in Supporting information folder. Scale bar = 50 μm. PI, propidium iodide. (JPG) [file pbio.3001454.s005.jpg]

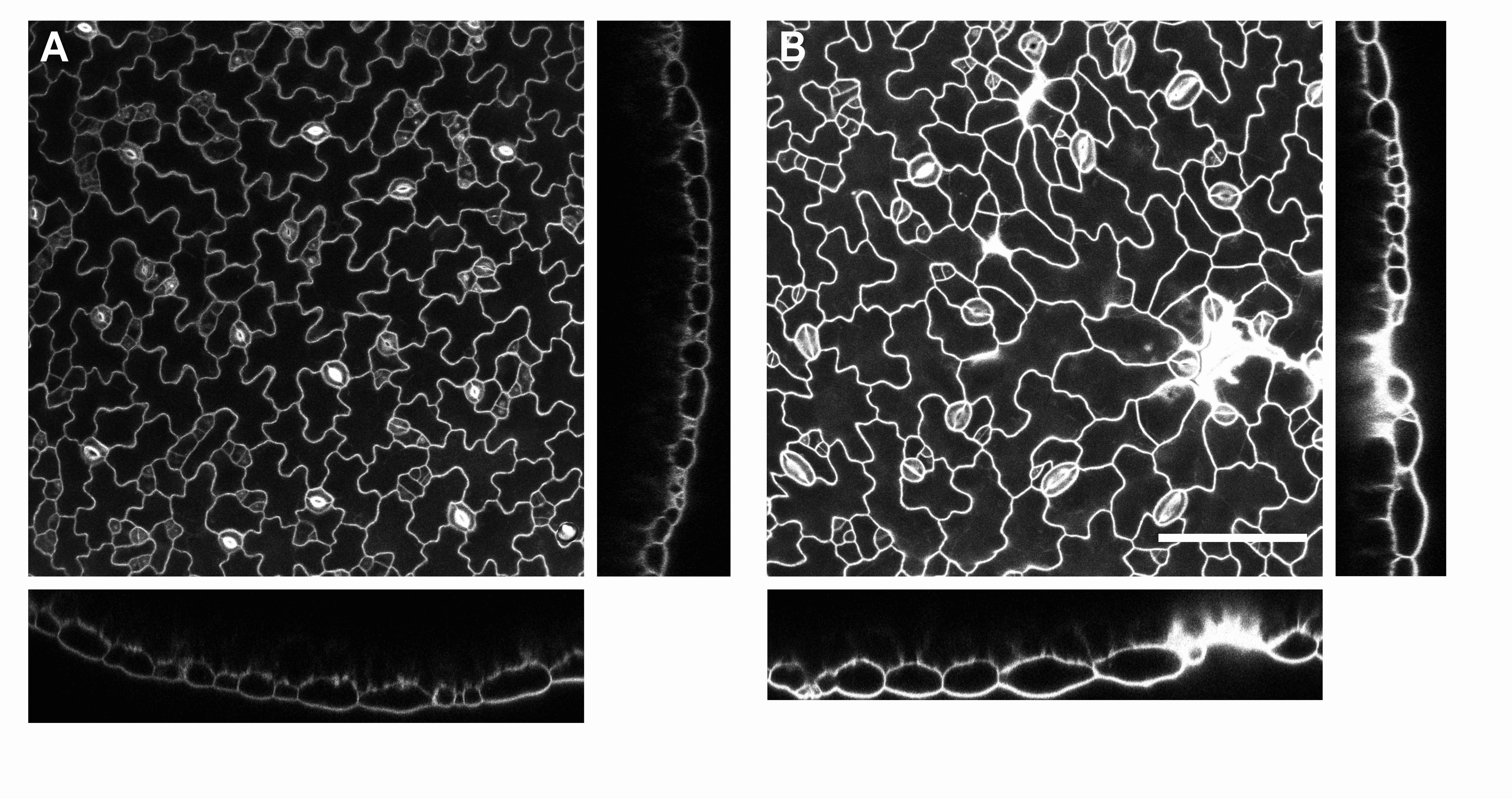

Supplement: S4 Fig — (A, B) Representative images of Col-0 (A) and fer-4 (B) pavement cells, from seedlings grown on a medium containing 0.7% agar at t = 4 DAG. Samples were PI stained and cell contours were extracted with SurfCut and projected in 2D. Orthogonal projections were also extracted with Fiji. Note the presence of curvy outer walls in some fer-4 cells. All underlying data can be found in Supporting information folder. Scale = 100 μm. PI, propidium iodide. (JPG) [file pbio.3001454.s006.jpg]

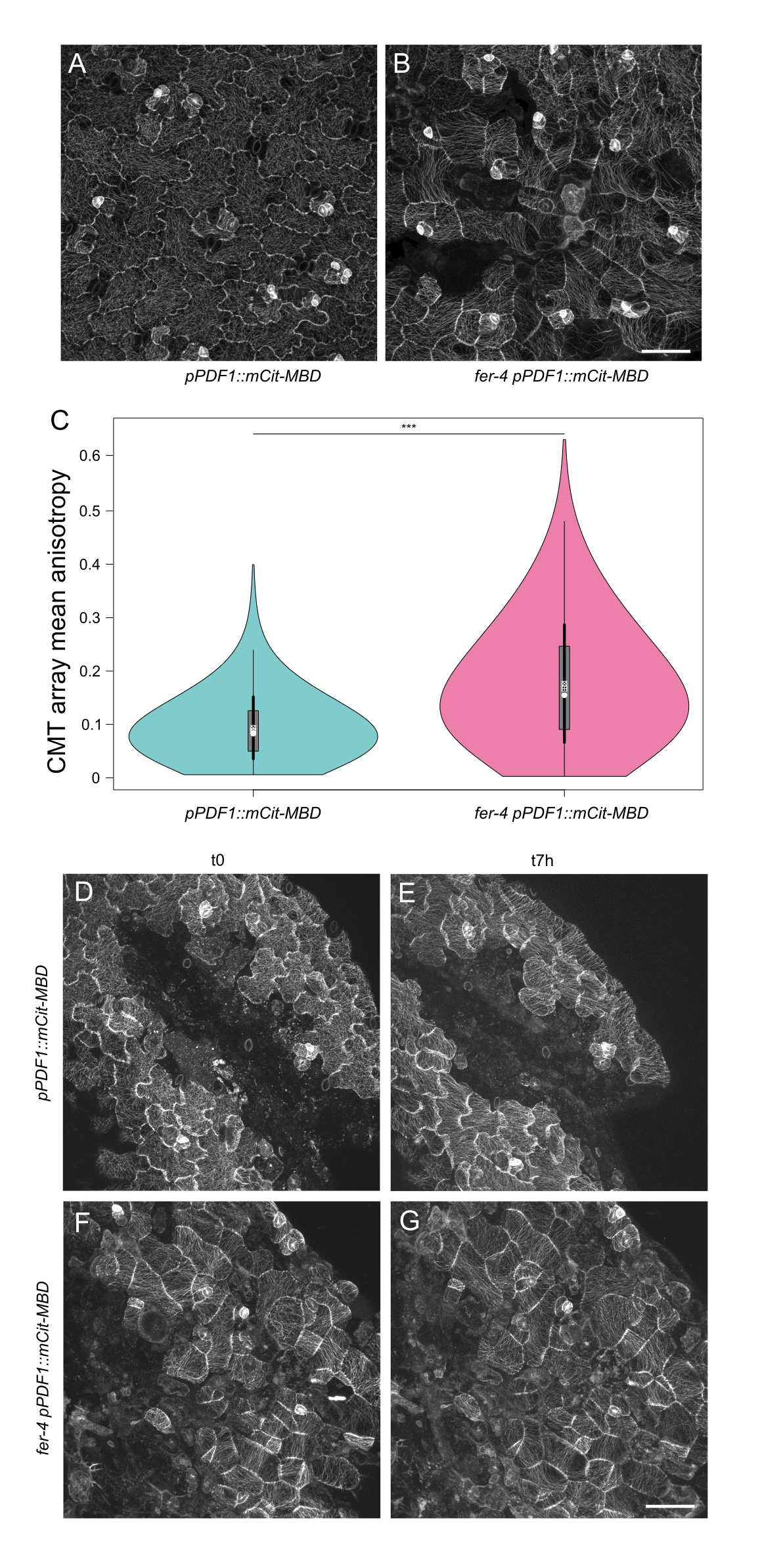

Supplement: S6 Fig — Representative confocal images of pPDF1::mCit-MBD (A) and fer-4 pPDF1::mCit-MBD (B) pavement cells from seedlings grown on a medium containing 0.7% agar. Quantification of the anisotropy of cortical microtubule arrays in both lines (C). Note the presence of circumferential cortical microtubules around dead cells, matching the predicted maximal tensile stress direction. Representative confocal images of pPDF1::mCit-MBD (D, E) and fer-4 pPDF1::mCit-MBD (F, G) pavement cells from seedlings grown on a medium containing 0.7% agar, immediately after ablation (t0, D, F) and 7 hours later (t7h, E, G). Note the presence of many dead cells and the strong alignment of cortical microtubules at t0 in fer-4. All underlying data can be found in Supporting information folder. Scale = 50 μm. (JPG) [file pbio.3001454.s008.jpg]

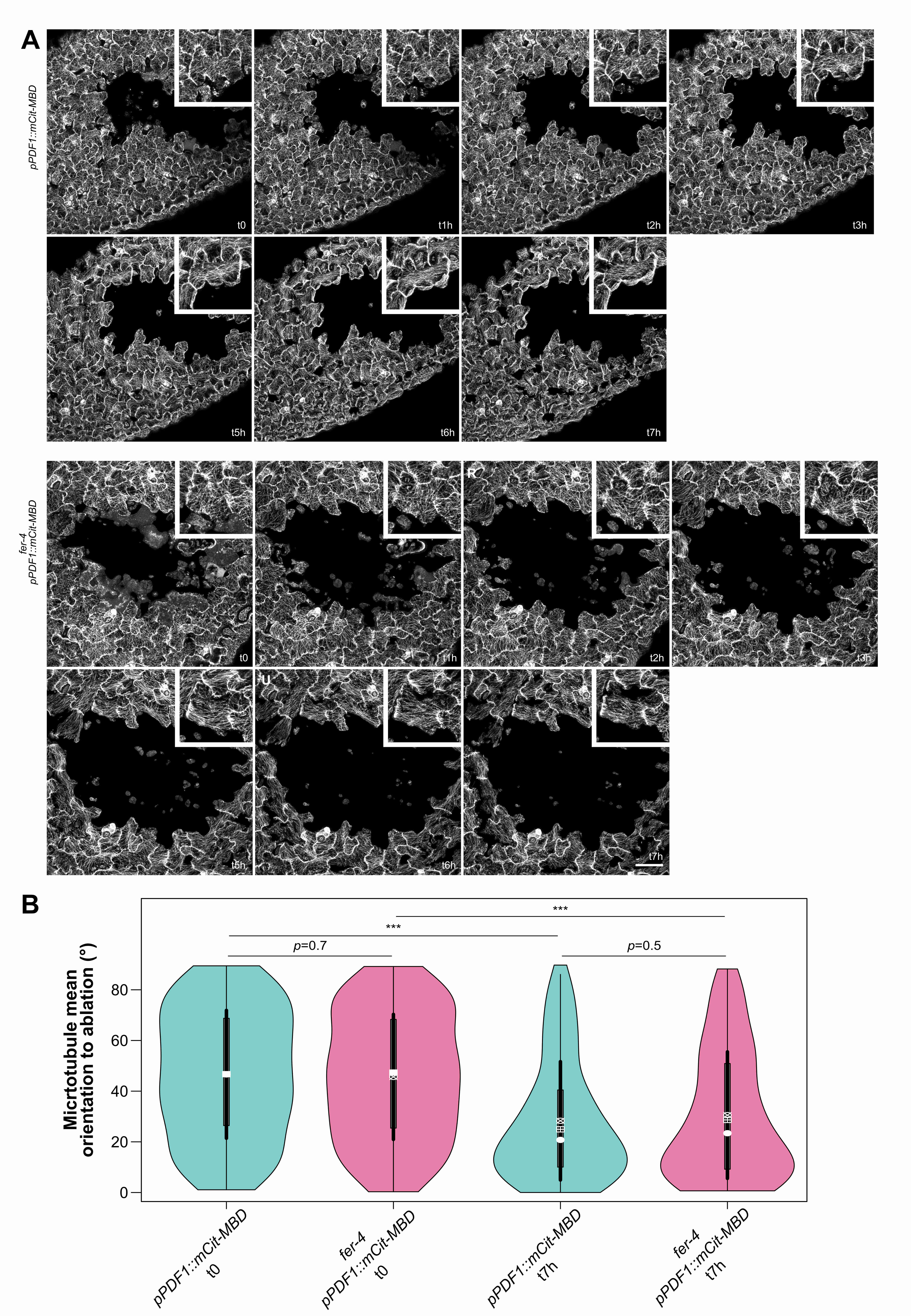

Supplement: S7 Fig — (A) Representative confocal images of pPDF1::mCit-MBD (top) and fer-4 pPDF1::mCit-MBD (bottom) pavement cells, from seedlings grown on a medium containing 2.5% agar, immediately after an ablation (t0) and 1 to 7 hours later. Scale = 50 μm. (B) Orientation to the ablation (violin plot) of cortical microtubule arrays and p-values of Wilcoxon–Mann–Whitney tests in cells surrounding the ablation site in pPDF1::mCit-MBD and fer-4 pPDF1::mCit-MBD pavement cells, immediately after an ablation (t0) and 7 hours later (t7h) (see Fig 4). All underlying data can be found in Supporting information folder. (JPG) [file pbio.3001454.s009.jpg]

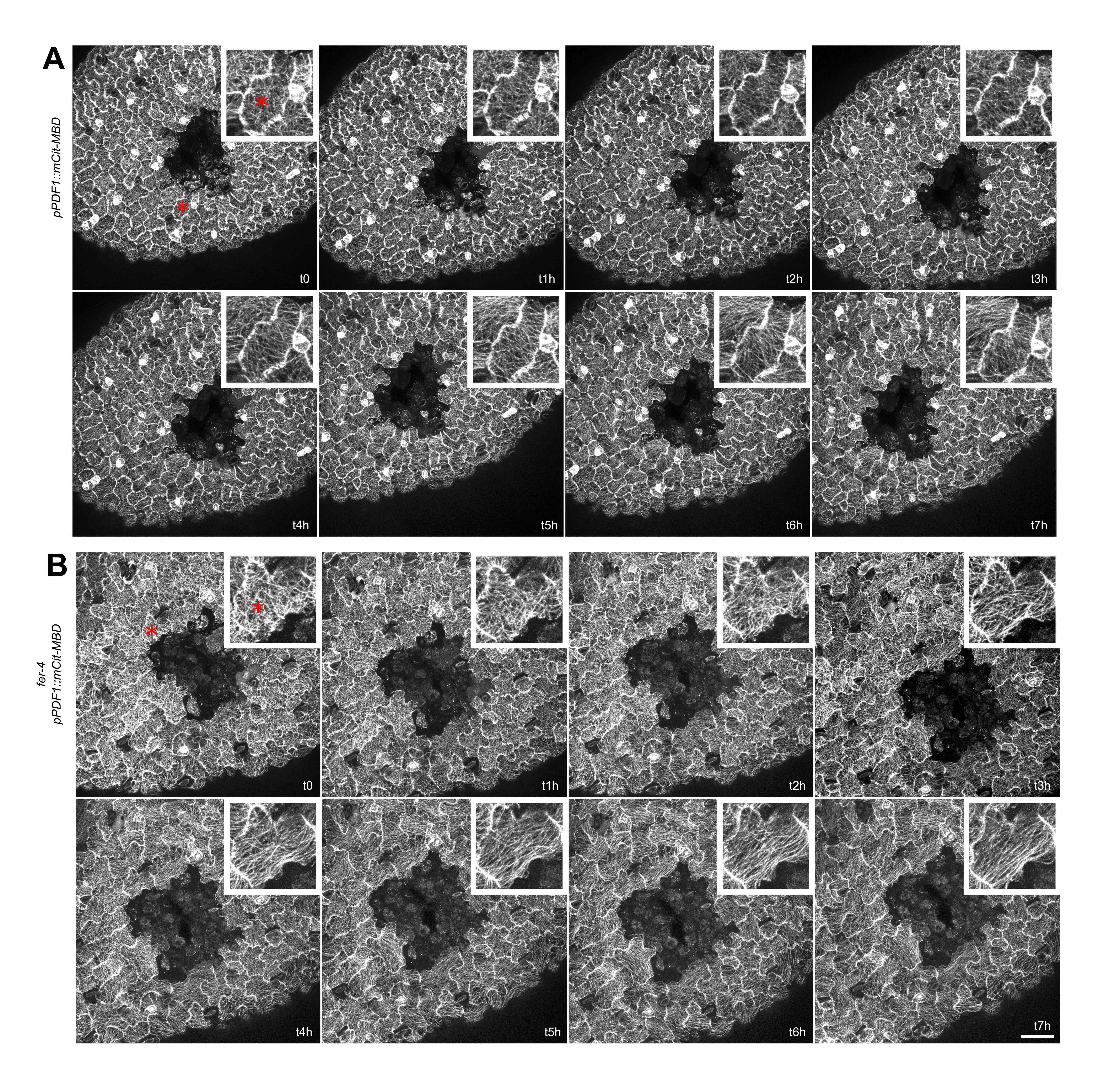

Supplement: S8 Fig — (A) Representative confocal images of pPDF1::mCit-MBD (A) and fer-4 pPDF1::mCit-MBD (B) pavement cells, from seedlings grown on a medium containing 2.5% agar, immediately after an ablation (t0) and 1 to 7 hours later. Scale = 50 μm. (JPG) [file pbio.3001454.s010.jpg]

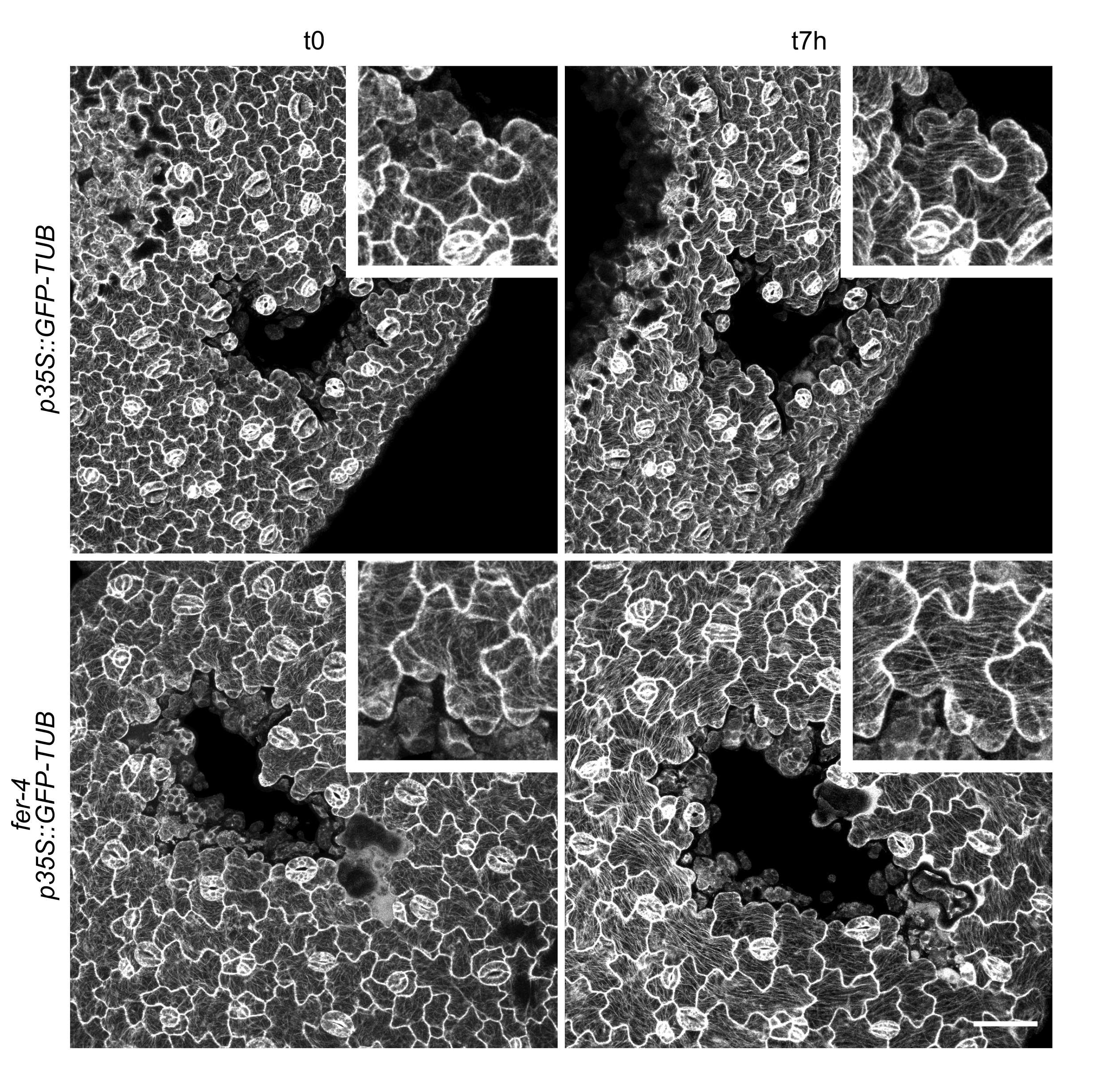

Supplement: S9 Fig — Representative confocal images of p35S::GFP-TUB (top) and fer-4 p35S::GFP-TUB (bottom) pavement cells from seedlings grown on a medium containing 2.5% agar, immediately after an ablation (t0) and 7 hours later (t7h). Scale = 50 μm. (JPG) [file pbio.3001454.s011.jpg]

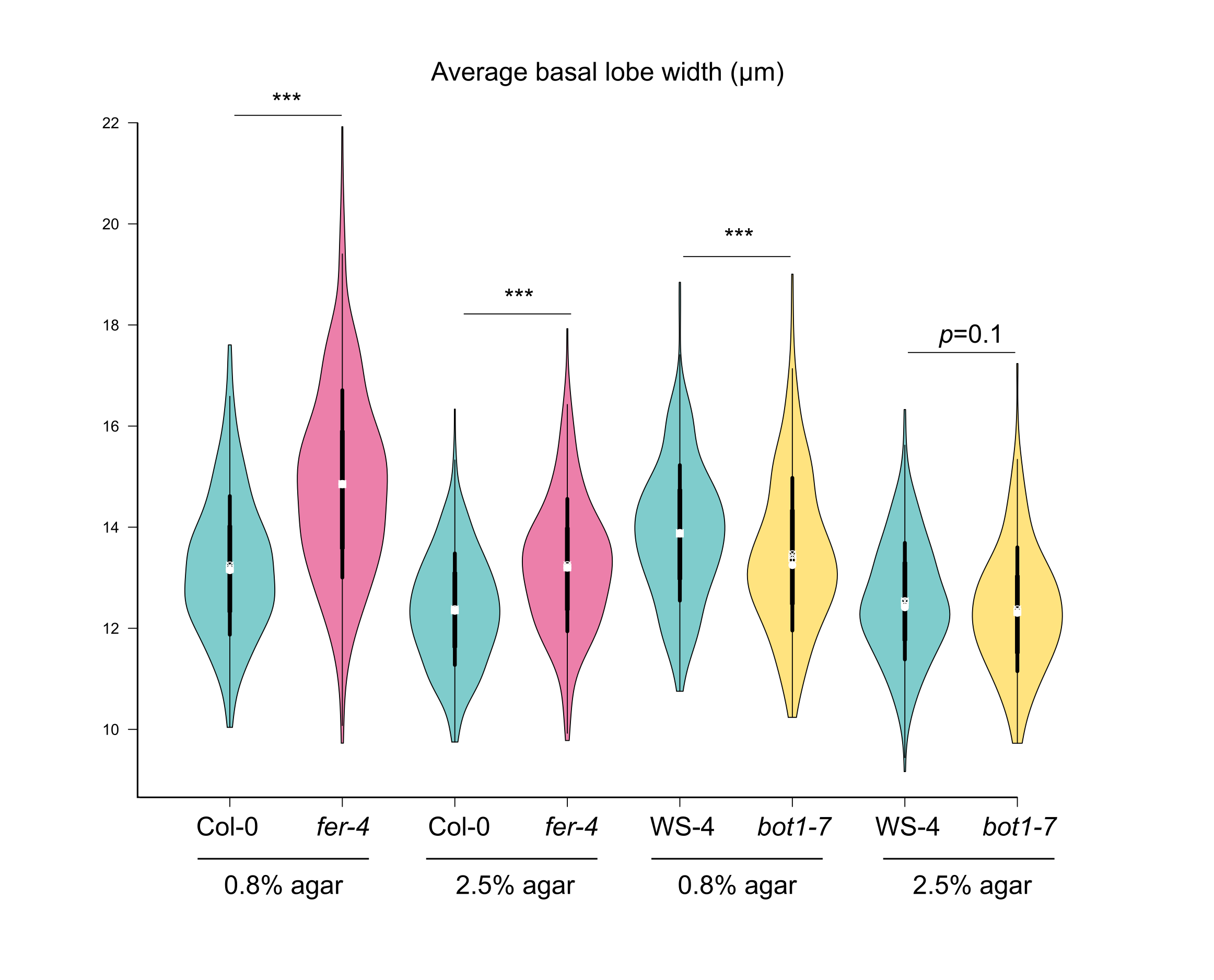

Supplement: S10 Fig — Basal lobe width (violin plot) of pavement cells and p-values of Dunn tests for the WT (Col-0, WS-4), katanin mutant (bot1-7), and fer-4. Seedlings were grown on 0.8% or 2.5% agar. Data from Fig 5A (0.8% agar) are reproduced here to allow a full comparison of the results. All underlying data can be found in S7 Data. WT, wild type. (PNG) [file pbio.3001454.s012.png]
